# Janus ScYCBr_2_ MXene as a Promising Thermoelectric Material

**DOI:** 10.1021/acsaem.4c01221

**Published:** 2024-07-22

**Authors:** Mounir Ould-Mohamed, Tarik Ouahrani, Reda Boufatah, Ángel Morales-García, Ruth Franco, Michael Badawi, Daniel Errandonea

**Affiliations:** † LPTHIRM, Département de Physique, Faculté des Sciences, Université Saâd Dahlab-Blida 1, B.P. 270 Route de Soumâa, Blida 09000, Algeria; ‡ Ecole Supérieure en Sciences Appliquées, ESSA-Tlemcen, BB 165 RP Bel Horizon, Tlemcen 13000, Algeria; § Laboratoire de Physique Théorique, Université de Tlemcen, Tlemcen 13000, Algeria; ⊥ Université de Lorraine, Laboratoire Lorrain de Chimie Moléculaire CNRS, L2CM, Metz F-57000, France; ∥ Laboratoire de Physique Théorique, Université de Tlemcen, Tlemcen 13000,Algeria; # Departament de Ciéncia de Materials i Química Física and Institut de Química Teórica i Computacional (IQTCUB), Universitat de Barcelona, c/Martí i Franquès 1-11, Barcelona 08028, Spain; ○ MALTA Consolider Team and Departamento de Química Física y Analítica, Universidad de Oviedo, Oviedo E-33006, Spain; △ Departamento de Física Aplicada - Instituto de Ciencia de Materiales, Matter at High Pressure (MALTA) Consolider Team, Universidad de Valencia, Edificio de Investigación C/Dr. Moliner 50 Burjassot, Valencia 46100, Spain

**Keywords:** Thermoelectric, Ab initio calculations, Seebeck effect, Thermal conductivity, Green energy

## Abstract

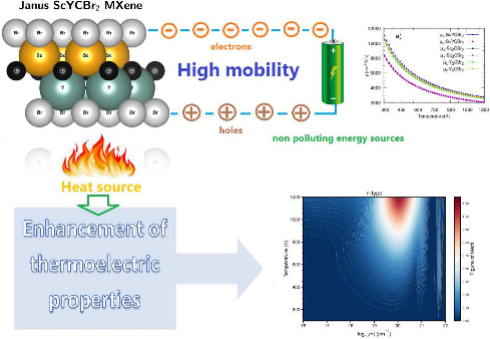

Finding green energy resources that contribute to the battle against global warming and the pollution of our planet is an urgent challenge. Thermoelectric electricity production is a clean and efficient method of producing energy; consequently, scientists are currently researching and creating thermoelectric materials to increase the efficiency of thermoelectric electricity production and expand the potential of the thermoelectric effect for clean energy production. This work focuses on a comprehensive study of the thermoelectric properties of two-dimensional ScYCBr_2_. We report here a computational analysis of this Janus-like MXene, which is predicted to exhibit outstanding thermoelectric properties. The study uses density-functional theory to provide evidence of the important role played by symmetry breaking to promote low-thermal transport by favoring certain phonon scattering channels. Compared to its symmetric parent compounds, the asymmetric Janus-type ScYCBr_2_ displays additional phonon scattering channels reducing the thermal conductivity. An exhaustive investigation of the dynamical stability for both zero-temperature and high-temperature conditions was also performed to support the stability of ScYCBr_2_. Our analysis shows that thanks to its asymmetric structure, the ScYCBr_2_ MXene has thermoelectric properties that largely surpass those of its parent symmetric counterpart Sc_2_CBr_2_, being a material with a remarkable thermoelectric high figure of merit. Another advantage of ScYCBr_2_ is its high carrier mobility. This work not only demonstrates that this material is a promising thermoelectric material but also shows that ScYCBr_2_ can operate efficiently at high temperatures up to 1200 K.

## Introduction

Thermoelectric materials (TEM) offer a green and sustainable way to produce electricity which could be beneficial for the planet compared to hydrocarbons and other contaminant sources of energy. Thermoelectric devices do not produce contaminant emissions, not contributing thus to global warming.^1^ TEM are ideal for constructing thermoelectric generators that allow heat to be directly transformed into electricity based on the Seebeck effect.^2^ Thermoelectric devices could be used in power plants and factories to convert waste heat into electrical power and in cars to increase fuel efficiency. One of the most important challenges for developing competitive thermoelectric technologies is creating highly efficient TEM, which would be very helpful for a variety of environmental applications. The performance of TEM materials is currently characterized by the dimensionless figure of merit (ZT) defined as ,^3^ where *S* stands for the Seebeck coefficient, σ for the electrical conductivity, *T* for the absolute temperature, and κ_*e*_ and κ_*l*_ for electronic and lattice thermal conductivity, respectively.

Two-dimensional (2D) materials have shown to be very promising for developing highly efficient TEM due to their high thermoelectric figures of merit.^4,5^ Thanks to this characteristic and their excellent mechanical properties they might be utilized to create flexible and ultrathin thermoelectric devices, which would satisfy the requirements of the developing nanoelectronics market.^6^ In addition, 2D materials have a large surface area-to-volume ratio, which gives them unique characteristics, including adjustable chemical functionality, and an electrical conductivity that in some cases is greater than that of their bulk counterparts.^7,8^ They are also appropriate for thermoelectric devices due to their reduced thermal conductivity and improved charge carrier mobility.^7,8^ Thanks to these characteristics, over recent years, 2D materials have gained large interest as high-performance TEM for efficient conversion of energy in small-packed power generators and cooling devices.^9^ The 2D materials explored for improving thermoelectric devices include graphene, transition metal dichalcogenides, and recently MXene compounds (MXenes).^10^

The emergence of 2D MXene structures in 2011^11^ has sparked significant research interest, making them a captivating area of study ever since. The basic configuration of 2D MXene compounds is described by the chemical formula M_*n*+1_X_*n*_T_*x*_^12^ (where *n* ranges from 1 to 3), where *M* represents an early transition metal, X denotes carbon and/or nitrogen, and T corresponds to surface terminations such as oxygen or fluorine. The MXene family has unique properties, including high mechanical strength, high electrical conductivity, and hydrophilicity. MXene materials also exhibit remarkable electrochemical properties, making them promising candidates for energy storage applications,^13,14^ such as batteries and supercapacitors. Additionally, MXenes have potential applications in catalysis,^15,16^ water purification,^17^ and electromagnetic interference shielding.^18^

Experiments reported by Zha et al*.*^19^ showed that the Ti_2_CO_2_ MXene exhibits promising characteristics for thermoelectric applications. More recently, while investigating the Pt_2_CO_2_ Mxene monolayer, Wei et al.^20^ measured a large *ZT* with a value approaching 1.05 at 1000 K, which supports the potential use of Pt_2_CO_2_ as a thermoelectric material. Moreover, using the Boltzmann theory, Kumar et al.^21^ have predicted remarkably high Seebeck coefficients for the MXene compounds Sc_2_CO_2_ and Sc_2_CF_2_, being their Seebeck coefficients as high as 1022 and 1036 μV/K at room temperature, respectively. Significantly, the Y_2_CF_2_ MXene exhibits a figure of merit of 1.38 at 900 K, which was attributed to its low thermal conductivity. This implies its potential application as a medium-temperature TEM.^22^ In summary, the growing family of 2D MXene materials offered exciting opportunities for developing clean sources of energy.

It is well-known that the structural characteristics of 2D materials lead to localization effects which favor an increased phonon scattering and a decreased thermal conductivity.^23^ Increasing the complexity of the crystal structure is an efficient way to increase the phonon scattering channels by breaking the degeneracy of phonon modes, therefore helping to effectively reduce the thermal conductivity. It has been conjectured that an asymmetric 2D structure derived from the MXene structure (for instance a Janus-type structure) might inherit the primary characteristics of the phonon dispersion of the MXene parent compounds. Nevertheless, the specific features of the phonon dispersion of the Janus structure, due to the broken symmetry, might reduce the lattice thermal conductivity of the asymmetric 2D structure. Derivation of Janus compounds from M_2_XT_2_ MXenes can be done in different ways as heterogeneity in the surfaces is possible through manipulation of the M as well as the T elements.^24^ Studies with complex structures employing Janus-like derived MXenes showed that the electric dipole in these structures is exceptionally high and perpendicular to the 2D plane, enhancing the phonon scattering rates,^25^ reducing the thermal conductivity, and consequently improving the thermoelectric response. Due to the fact that charge redistribution influences the electrical conductivity of materials, breaking the symmetry of the crystal structure can also enhance the electrical conductivity by introducing additional charge carriers (electrons and/or holes), which are critical for the efficiency of thermoelectric materials. So the introduction of an asymmetry in the crystal structure could enhance the transport properties. The asymmetric structure also gives rise to an intrinsic dipole moment, leading to a band edge realignment.^26^ This can result in preferential scattering directions or modified carrier mobilities, impacting in the electrical and thermal conductivity. The anisotropy of the material also influences carrier scattering mechanisms and promotes the formation of energy-dependent scattering mechanisms which enhance the Seebeck coefficient (*S*).^27^ This effect arises from the asymmetry-induced modifications in the electronic density of states near the Fermi level, affecting the thermoelectric power generation capacity and modifying selection rules that determine allowed and forbidden scattering channels in a given material.^28^ Additionally, the dipole moments can introduce additional scattering mechanisms for phonons. When phonons encounter polar molecules or ions with dipole moments, they experience an interaction that can scatter phonons, reducing their mean free path and thus lowering the thermal conductivity.

To understand the influence of asymmetry on thermoelectric properties, we examine here the asymmetric Janus-type ScYCBr_2_ MXene, evaluating its thermoelectric characteristics in relation to its parent symmetric compound Sc_2_CBr_2_. Y_2_CBr_2_ was also studied but it was found to be dynamically unstable being therefore excluded from the discussion of the results. We will show that the effects of the asymmetry induced by the presence of different atoms in the surfaces (Sc or Y, see Figure S1 in the Supporting Information) mainly affect the high-order phonon scattering, which allows for the regulation of thermal conductivity via anharmonic interactions. In particular, we have found noticeably differences in the behavior of the three low-frequency flexural acoustic phonons, which highly influence the behavior of the thermal conductivity.^29^ In addition, we will present results on the structural stability, and the phonon transport and electronic transport properties of the studied compound.

## Computational Methodology

In the present work, spin-polarized first-principles calculations have been conducted using the density-functional theory (DFT).^30^ The computer simulations have been carried out using the Quantum Espresso (QE) package.^31^ The gradient-corrected exchange-correlation functional of the Perdew–Burke–Ernzerhof for solids (PBEsol) form has been adopted to describe the exchange-correlation energy within the general-gradient approximation (GGA).^32^ The DFT-D2 approach elaborated by Grimme^33^ has been employed to treat the dispersion interactions. We have used an 8 × 8 × 1 grid of Monkhorst–Pack **k**-points^34^ for sampling the Brillouin zone. The kinetic-energy cutoff for the wave function and charge density have been respectively taken to be equal to 45 and 360 Ry. The electronic convergence criteria have been set to 10^–10^ Ry. Also, a Marzari-Vanderbilt scheme has been used with a 0.01 Ry width. The atomic positions in the unit cell and the lattice parameters have been relaxed by using the variable-cell command of the QE package based on the Broyden-Fletcher-Goldfarb-Shanno procedure^35^ until the forces on each atom were converged and the force on each atom reached less than 7.49 × 10^–6^ Ry/a.u. Due to the two-dimensional nature of the studied structure, a vacuum space of 25 Å along the *z* – direction has been applied. This permits us to eliminate interactions between neighboring slabs. Additionally, the Heyd-Scuseria-Ernzerhof (HSE06) hybrid functional^36^ has been used to calculate the electronic properties. This approach allows a more accurate calculation of the electronic band structure and carrier mobility overcoming the limitations of the traditional PBEsol functional. The phonon dispersion curves, the corresponding phonon density of state, phonon group velocities, and harmonic force constants have been calculated with a supercell of 2 × 2 × 1 using the finite displacement method. Long-range electrostatic interactions (nonanalytical correction) are included by using the Born effective charges. These calculations have been performed by using both Phonopy^37^ and Alamode^38^ packages interfaced with the Quantum Espresso code. The linearized phonon Boltzmann equation (BTE) has been employed to compute the lattice thermal conductivity (κ_*L*_). This calculations has been carried out employing the single-mode relaxation time approximation (RTA) as incorporated in the Phono3py package interfaced with the QE code, In both codes, the expression for κ_*L*_ is formulated as follows:
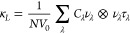
1where *N*, *V*_0_, and λ are the total number of **q** points used for sampling the Brillouin Zone, the volume of the unit cell, and the phonon mode respectively, while *C*_λ_, ν_λ_, and τ_λ_ refer to specific heat, phonon group velocity, and phonon lifetime, respectively. In these calculations, the finite-displacement method has been employed to calculate the second-order force constants (harmonic contribution). Such calculations have been carried out on a 2 × 2 × 1 supercell sampled with a dense 8 × 8 × 1 **k**-point mesh. The third-order and four-order anharmonic interatomic force constants (IFCs) were calculated using the finite displacement method with the same supercell (convergence tests are displayed in Figure S2 of the Supporting Information). For the computation of the lattice thermal conductivity, the reciprocal space of the primitive cell has been sampled by a 50 × 50 × 1 **k**-point meshes. The lattice thermal conductivity of 2D materials is influenced by the unit-cell length along the **z**-direction. Normalization is then essential. It can be achieved by multiplying these values by the ratio *L*_*z*_/*d*,^39^ where *L*_*z*_ represents the unit-cell length along the **z**-direction and *d* is the effective thickness (this magnitude is obtained as the sum of the atomic layer thickness and the van der Waals radius of the atoms of the surfaces of the MXene structure^41^). To compute the anharmonic phonon dispersion as a function of temperature, the fourth-order force constants using the harmonic eigenvectors should be computed. This calculation is not implemented in Phonopy but is implemented in the Alamode code^38^ which has been used to calculate the harmonic and anharmonic interatomic force constants in order to evaluate the anharmonic phonon dispersion (for more details see the Supporting Information). Finally, the electronic transport properties of the Janus-type ScYCBr_2_ MXene, including the Seebeck coefficient and electrical and thermal conductivity, have been computed using the BoltzTrap2 code^42^ interfaced with the QE software.

## Results and Discussions

**Figure 1:**
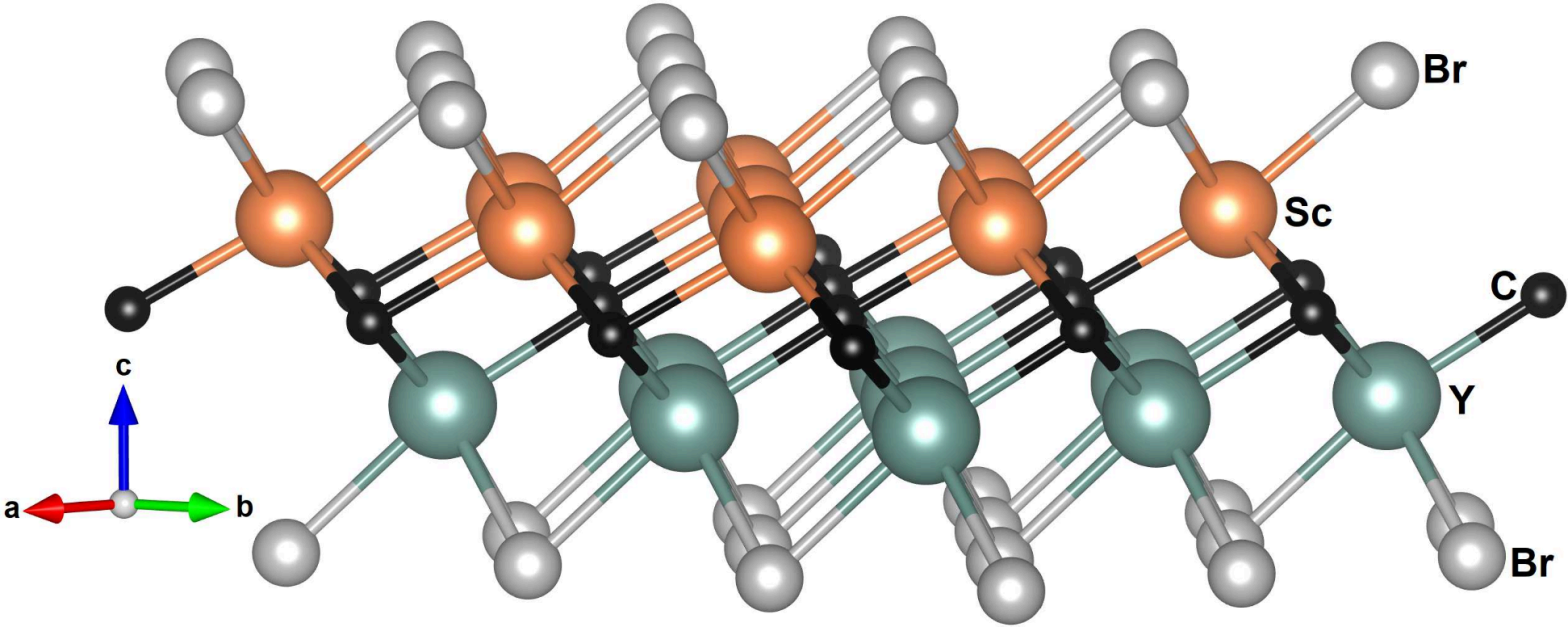
Schematic representation of the Janus ScYCBr_2_ MXene. Gray, dark yellow, black, and metallic green spheres represent Br, Sc, C, and Y atoms, respectively.

2D Sc_2_CBr_2_ has a noncentrosymmetric trigonal layered crystal structure described by the space group *P*3*m*1 (No. 164) and has a point group symmetry *D*_3*d*_ (−3m). 2D ScYCBr_2_ has an asymmetrically ordered Janus-type structure^43,44^ described by space group *P*3*m*1 (No. 156) and has a point group *C*_3*v*_ (3m). It is formed by five atomic layers arranged in the sequence Br–Sc–C–Y–Br. In contrast Sc_2_CBr_2_ is a symmetric MXene structure. The asymmetry of ScYCBr_2_ allows its two surfaces to have different physical properties. The atomic arrangement of ScYCBr_2_ is shown in Figure 1 where we show a perspective view of the 2D structure. In terms of structural characteristics as well as the cluster expansion (CE) method,^24^ the Br atomic layers of ScYCBr_2_ MXenes can occupy either the *hcp-hcp*, *hcp-fcc*, *fcc-hcp*, or *fcc-fcc* hollow sites (See the four configurations in Figure S3 of the Supporting Information). However, after structural optimization, we have found that the *hcp-hcp* occupation gives the most stable configuration. The total energy per atom of the four configurations are respectively, E(*hcp-hcp*) = −8.24 eV/atom, E(*hcp-fcc*) = −8.18 eV/atom, E(*fcc-hcp*)= −8.14 eV/atom and E(*fcc-fcc*) = −8.08/atom eV. Thus, the functional groups prefer to occupy hollow sites that are associated with the Sc/Y sites stabilizing them in an *hcp-hcp* configuration. The optimized lattice constants, bond distances, and thickness of the more stable *hcp-hcp* configuration are summarized in Table 1. The table includes the lattice parameters previously reported for ScYCBr_2_ and Sc_2_CBr_2_ and related compounds.^45−47^ In contrast to the parent structures, in the Janus-type structure, the bond lengths of Sc–Br and Y–Br are different. According to the calculated thickness, we can expect that the investigated compound has pronounced quantum-confinement (QC) effects.^48^ Due to the dimension of the thickness, phonons will play a major role in thermal transport. However, because of the strong temperature dependence, the role of phonons declines gradually in the presence of a temperature gradient, which is mainly induced by the suppression of the phonon lifetime.

**Table 1:** Optimized Lattice Constant (*a* (Å)), Thickness of the 2D Layer (Δ*h* (Å)), and Bond Distances (*d* (Å)) of ScYCBr_2_ and Sc_2_CBr_2_ 2D Structures Compared to Previous Results on Compounds of the Same Family^a^

	*a*	Δ*h*	*d*_Sc–Y_	*d*_Sc–*C*_	*d*_Sc–Br_	*d*_Y–Br_	*d*_Y–C_
ScYCBr_2_^∗^	3.565(1)	6.273(1)	3.2674(1)	2.381(1)	2.715(1)	2.845(1)	2.456(1)
Sc_2_C Br_2_^∗^	3.452(1)	6.038(1)	-	2.329(1)	2.694(1)	-	-
ScYCBr_2_^45^	3.650(1)	-	-	-	-	-	-
Sc_2_CBr_2_^46^	3.507(1)	-	-	-	-	-	-
Sc_2_CCl_2_^47^	3.470(1)	-	-	-	-	-	-
Y_2_CCl_2_^47^	3.520(1)	-	-	-	-	-	-

a The symbol * identifies the present results.

**Figure 2:**
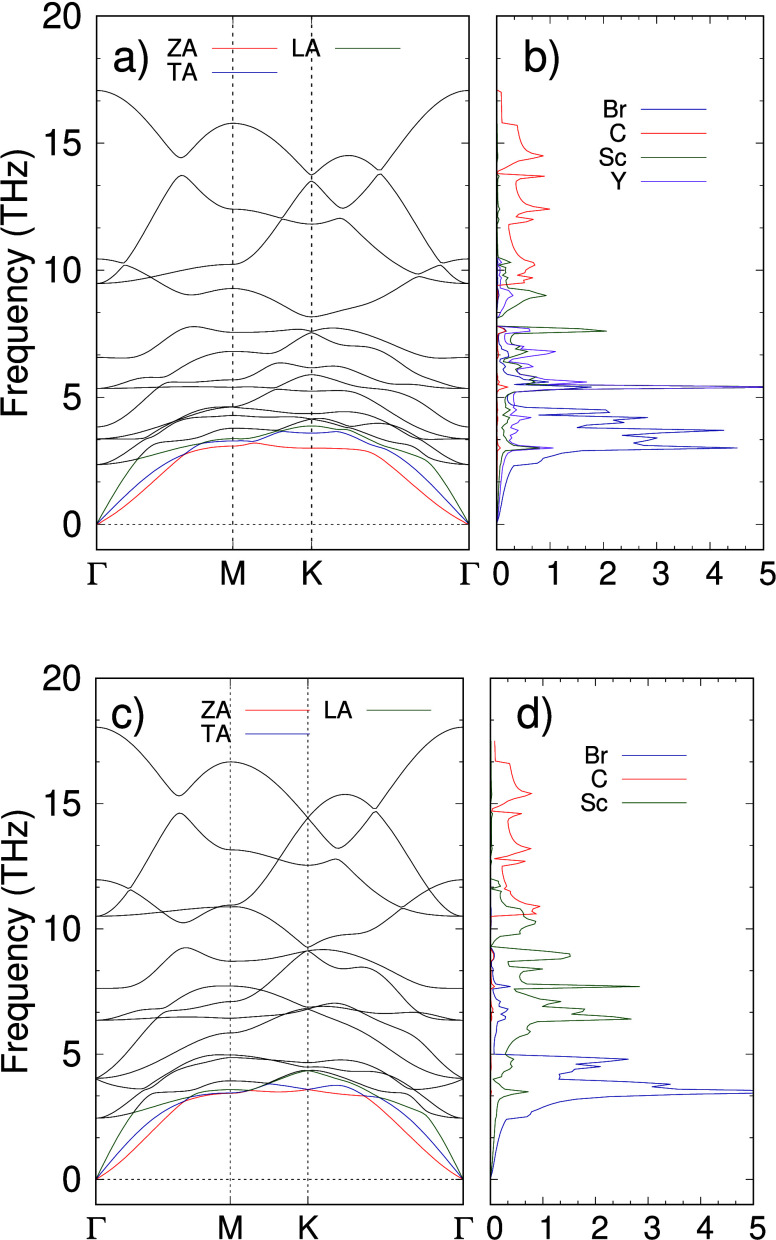
(a) Phonon dispersion and (b) density of states calculated at 0 K for ScYCBr_2_. (c) Phonon dispersion and (d) density of states calculated at 0 K for Sc_2_CBr_2_. The acoustic branches are shown in red (ZA mode), blue (TA mode), and green (LA mode).

To assess the dynamical stability of the investigated 2D structures, we have calculated the phonon dispersion for each compound. From them, we have obtained the phonon density of states (PHDOS). The phonon dispersion and PHDOS of ScYCBr_2_ are shown in Figure 2a and 2b. These calculations were carried out independently using the Phonopy and Almode codes, see Figure S4 in the Supporting Information. The agreement between the two methods suggests that both codes give similar results for the phonons of the investigated compounds. The phonon dispersion and PHDOS of Sc_2_CBr_2_ are shown in Figure 2c and 2d. Considering that the primitive cells of the studied compounds have five atoms, 15 phonon modes are expected, as illustrated in Figure 2. No imaginary phonon frequencies are observed for the two compounds, supporting their dynamical stability. We would like to add here that mechanical stability is also fulfilled (see Supporting Information and Table S1). The modes at the Γ point of the Brillouin zone can be labeled by irreducible representations as follows: for Sc_2_CBr_2_, M = 2*A*_1*g*_ + 3*A*_2*u*_ + 3*E*_*u*_ + 2*E*_*g*_, where Γ_*acoustic*_ = *A*_2*u*_ + *E*_*u*_ and Γ_*optic*_ = 2*A*_1*g*_(*R*) + 2*A*_2*u*_(*IR*) + 2*E*_*u*_(*IR*) + 2*E*_*g*_(*R*). For ScYCBr_2_, M = 5*A*_1_ + 5*E*, where Γ_*acoustic*_ = *A*_1_ + *E* and Γ_*optic*_ = 4*A*_1_(*R*/*IR*) + 4*E*(*R*/*IR*). Where the letters R and IR in the parentheses indicate whether the mode is Raman or infrared active, respectively. The parent structure possesses a higher symmetry than the Janus one. The break of the mirror symmetry^49^ leads to additional phonon branches in the Janus structure. This difference in crystal symmetry will be the cause of some restrictions on possible phonon scattering channels.^50^ This fact indirectly affects phonon scattering through energy and momentum conservation conditions.^51^ Thus, the broken in-plane crystal symmetry will impact the thermal conductivity.

We additionally studied the thermal stability of both compounds through *ab initio* molecular-dynamics (AIMD) calculations at a finite temperature to understand their response to thermal fluctuations. Figure S5 in the Supporting Information provides information to understand the response of the studied materials to thermal vibrations at ambient and high temperatures. The ScYCBr_2_ and Sc_2_CBr_2_ MXenes show periodic temperature fluctuations as well as a negative stable periodic total energy as a function of the AIMD time simulation.

**Figure 3:**
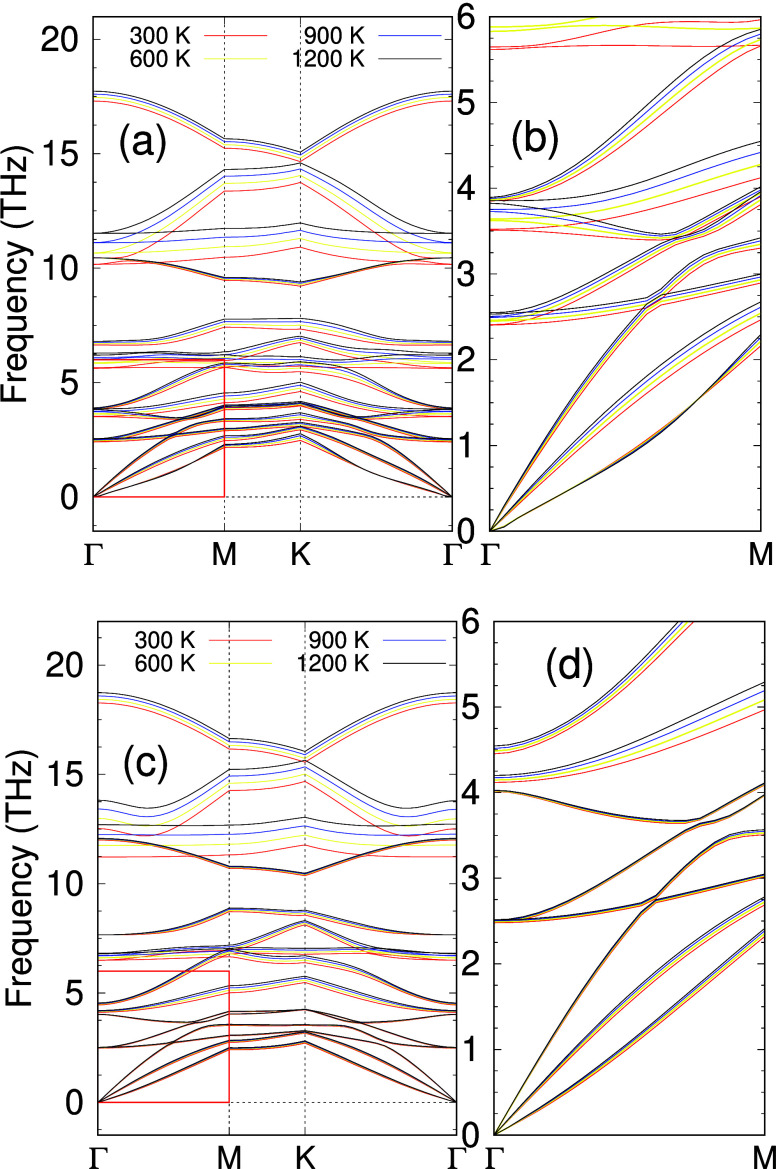
Anharmonic phonon dispersion curves as a function of temperature of (a) Janus-like ScYCBr_2_. In (b) we represent a zoom of the highlight region in the Γ – *M* direction. (c) and (d) show the same results for the ScCBr_2_ MXene.

From the phonon dispersions represented in Figure 2, it can be seen that, for both compounds, near the Γ point the dispersion relation of the transverse acoustic (TA) and longitudinal acoustic (LA) branches is linear. On the other hand, the out-of-plane flexural acoustic (ZA) branch exhibits a quadratic dispersion, which is a known characteristic of 2D materials. The nonlinearity is more pronounced in the case of the Janus-like ScYCBr_2_ MXene due to the asymmetric nature of the structure and the lack of mirror symmetry. Thus, the ZA mode is not a completely out-of-plane vibration as in Sc_2_CBr_2_, but one with an in-plane component, which causes the ZA mode to get involved in more scattering processes. Contrary to conventional symmetric chalcogenide monolayers, in Mxenes there is a splitting between the optical LO and TO modes at the Γ point^52^ in the phonon spectra which is caused by the dipole moment induced by the large electronegativity of Br. Regarding atomic contributions to vibrations, the carbon atoms mostly contribute to the optical branches in the high-frequency area; in contrast, the contributions of Sc and Y atoms dominate the intermediate frequency range and the contribution of Br atoms is dominant in the low-frequency region. The separation between vibrations associated with Y and Sc atoms in ScYCBr_2_ opens additional ways to control thermal conductivity by modifying the harmonic/anharmonic phonon characteristics due to the decoupling of their atomic vibrations and the unequal contribution of the different phonons to thermal transport at different frequencies.^29^ It is also important to note that according to the anharmonic phonon dispersions, here calculated at various temperatures (see Figure. 3a, 3b, 3c, and 3d), both ScYCBr_2_ and Sc_2_CBr_2_ are stable not only at 0 K but also at high temperatures from 300 to 1200 K. This fact indicates that ScYCBr_2_ and Sc_2_CBr_2_ deserve to be investigated as candidate materials to achieve high thermoelectric efficiency since often practical applications involve high temperatures.

**Figure 4:**
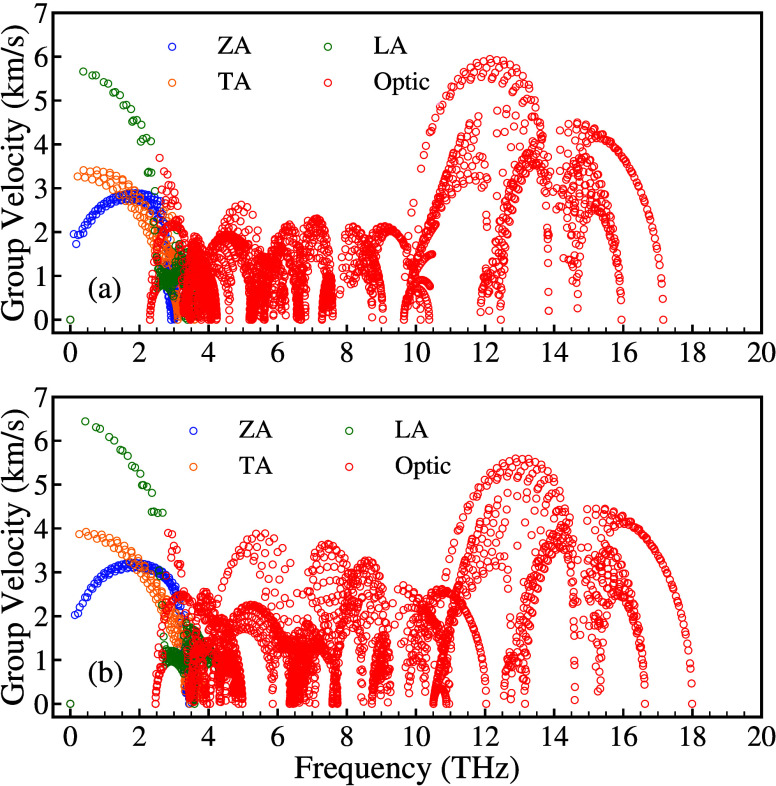
Calculated phonon group velocity of the (a) Janus-like ScYCBr_2_ MXene and (b) ScCBr_2_ MXene.

The phonon group velocity of each mode is given by , where *w*, *k*, and *q* represent the vibrational frequency, the vibrational mode index, and the wave vector, respectively. The group velocities of the three acoustic branches ZA, TA, and LA of ScYCBr_2_ near the Γ point are 2.82, 3.43, and 5.67 km/s, respectively (see Figure 4a). The group velocities are ordered following the sequence LA  TA  ZA. The ZA mode has a lower group velocity than the TA and LA modes, which is due to the nonlinear dispersion of the ZA branch near the Γ point. Consequently, the LA mode is responsible for the majority of the thermal transport. Analogous findings have been found in other 2D materials, including XTeI (X = Sb, Bi),^40^ SnY_2_ (Y = S,Se),^53^ and PbTe.^54^

It is noteworthy that the maximum of phonon group velocity plots is between 2.50 and 8.00 Km/s, which is typical in thermoelectric materials.^55^ The maximum phonon group velocity in ScYCBr_2_ is slightly smaller than 6 km/s, which is similar to the maximum phonon group velocity of several 2D materials, such as ZrOSe (6.1 km/s),^56^ PtAs_2_ (6 km/s)^57^ and TiS_3_ (6.5 km/s).^58^ Despite the differences between the crystal structures, ScYCBr_2_ and Sc_2_CBr_2_ have a similar group velocity for the ZA modes. Consequently, the specific heat (*C*_*v*_) curves are similar (see Figure S6 in the Supporting Information). The values of the specific heat of both compounds are low, of the order of 190 J/K, which fulfills a prerequisite for high-performance TE materials. According to the dynamical theory, the thermal conductivity is . Then a reduced group velocity and reduced *C*_*v*_ favor a low lattice thermal conductivity. Noticeably, compared to state-of-the-art thermoelectric asymmetric monolayers like SnSSe and PbSSe,^59^ whose acoustic mode occupies a wavenumber range of 0–3.5 THz, the acoustic phonon frequencies range of the Janus-like ScYCBr_2_ extends only up to 2.9 THz. In contrast, in ScCBr_2_ the range covered by the frequencies of the acoustic phonon modes is above 4 THz (see Figure 4b). This characteristic implies that the overall rate of the thermal energy transport in the Janus-like ScYCBr_2_ MXene is smaller than in the other materials of the same family. As a consequence of it, the heat conduction decreases, improving the ability of ScYCBr_2_ to convert temperature gradients into electrical power.

**Figure 5:**
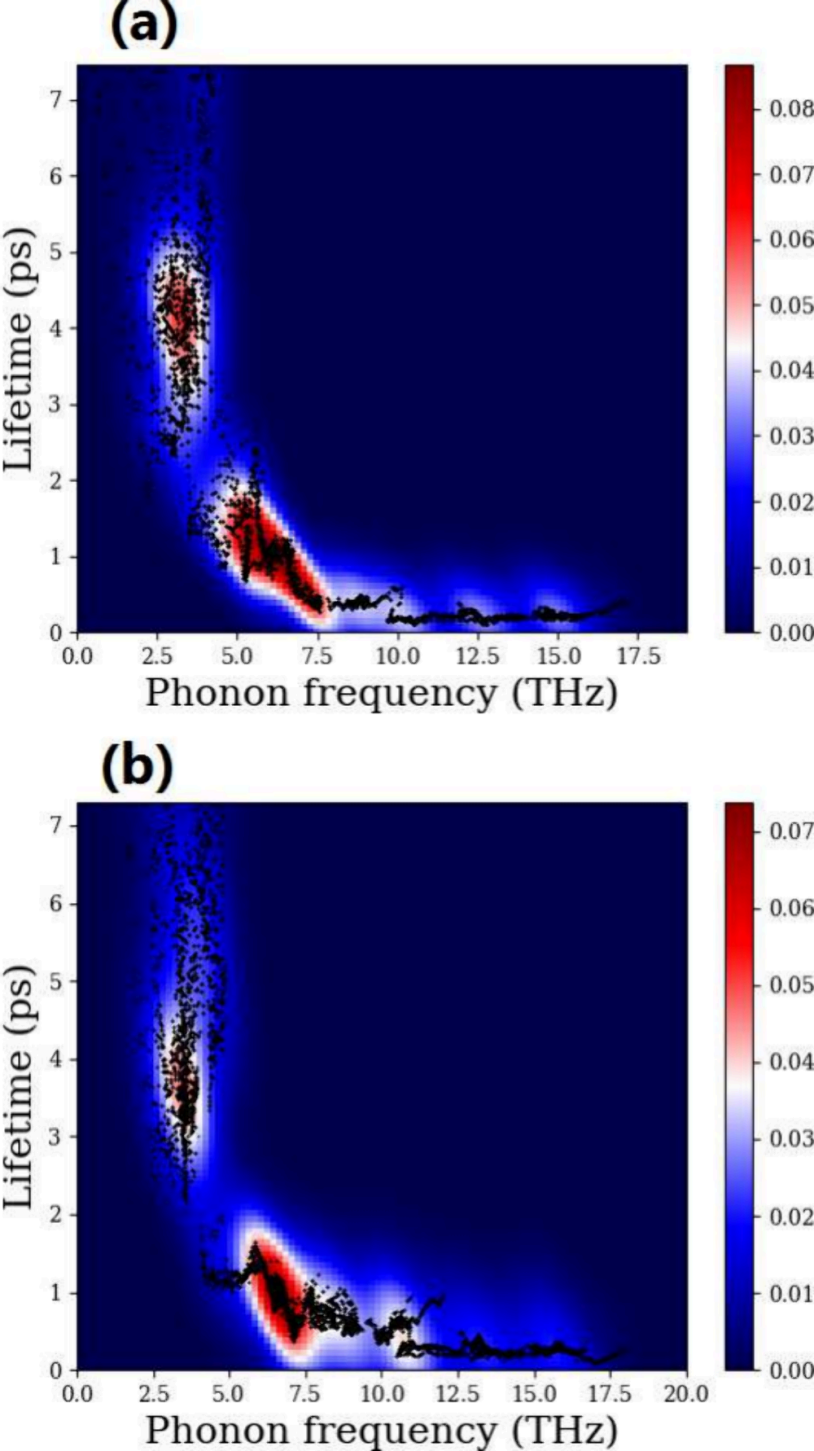
Phonon lifetime distribution vs frequency in (a) Janus-like ScYCBr_2_ MXene and (b) Sc_2_CBr_2_ MXene.

**Figure 6:**
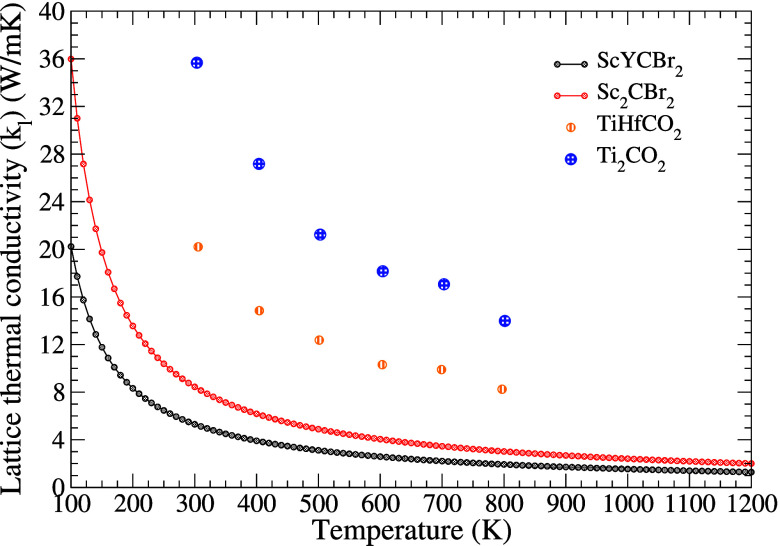
Calculated lattice thermal-conductivity κ_*L*_ of ScYCBr_2_ and Sc_2_CBr_2_. The plot also shows results from TiHfCO_2_ and Ti_2_CO_2_ taken from the work by Murari et al.^64^

To gain a deeper insight into the anharmonic properties of the phonons of ScYCBr_2_ and Sc_2_CBr_2_, we have also calculated the phonon lifetimes. Their values at room temperature are represented in Figures 5a and 5b. The lifetimes of the optical phonons of ScYCBr_2_ are considerably shorter than the lifetimes of the acoustic modes, going from 0.5 to 5 ps, and are shorter compared to the lifetime of the optical modes of SnSe,^60^ indicating that the optical modes have little contribution to the lattice thermal conductivity in ScYCBr_2_ (see Figure 5). More significantly, as shown in Figure 5a and 5b, the lifetime range determined for the Janus-like material is smaller than that of its parent Sc_2_CBr_2_ Mxene. To further explore the phonon modes responsible for the flow of heat within the material (heat carriers) on the two studied structures, we have investigated the contribution of different modes to κ_*L*_. Figure 6 shows the thermal conductivity of the two MXenes. It can be seen that ScYCBr_2_ has a lower thermal conductivity than its parent symmetric MXene and other MXenes. Notice that the inverse T dependence of κ_*L*_ suggests that to effectively address the phonon scattering, the Umklapp process needs to be taken into account.^61^ Additionally, the decrease of κ_*L*_ with increasing temperature is primarily attributed to the enhancement of phonon–phonon scattering with increasing temperature.

According to our calculations, the room-temperature thermal conductivity of ScYCBr_2_ is 5.24 Wm^–1^K^–1^ and it decreases to 1.27 Wm^–1^K^–1^ at 1200 K. On the other hand, in Sc_2_CBr_2_ κ_*L*_ is 8.44 W m^–1^ K^–1^ at ambient temperature. These values are significantly lower than the thermal conductivity of graphene (2200 Wm^–1^K^–1^), WS_2_ (72 Wm^–1^K^–1^),^62^ and WSe_2_ (53.0 Wm^–1^K^–1^),^63^ and much lower than the thermal conductivity of similar structures like TifCO_2_ and Ti_2_CO_2_ which are reported in the study performed by Murari et al.^64^ To understand the atomic contribution to κ_*L*_, we have analyzed it using the cumulative lattice thermal conductivity as a function of the phonon frequency. This analysis gives the primary causes of such low κ_*L*_. As can be seen in Figure S7 in the Supporting Information, in ScYCBr_2_ the modes below 3 THz account for 70% of the lattice thermal conductivity. According to the PHDOS, the main contribution to heat transport comes from the vibrations of Br atoms (see Figure 2).

The interactions between phonons and defects, impurities, and other phonons determine the transport characteristics of phonons in materials, including the thermal conductivity. In particular, the thermal conductivity depends on the balance between phonon scattering mechanisms and phonon transport, Strong scattering processes (e.g., due to high defect concentrations or rough interfaces) result in a low thermal conductivity, whereas materials with few scattering centers exhibit a high thermal conductivity. So, we will analyze these processes in the two studied materials.

Combining the linearized phonon BTE and the law of Fourier, the lattice thermal conductivity (κ_l_) can also be expressed as^65^
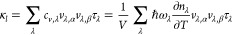
2where α and β are the Cartesian coordinates, λ ≡ (**q**, *v*) denotes the phonon mode with wave vector **q** and phonon polarization *v*, *c*_*v*,λ_ is the phonon specific heat capacity, *v*_λ_ is the phonon group velocity, and τ_λ_ is the phonon relaxation time. *V* is the volume of the primitive cell, *ℏ* is the reduced Planck constant, ω_λ_ is the phonon frequency, and *n*_λ_ is the Bose–Einstein distribution at temperature *T*. Getting τ_λ_, which is connected to multiple scattering processes, is a crucial in the described calculations. To avoid the boundary scattering effects, in our calculations, we have used the relation 1/τ_λ_=  +  + , which assesses the total relaxation time in RTA and it is obtained from the isotopic and anharmonic three-phonon scattering processes. Figure S8 in the Supporting Information presents the anharmonic three-phonon (3ph), four-phonon (4ph), isotopic, and also the sum of the 3 ph and 4ph contributions. According to our results, the 3ph-phonon mechanism is the major contribution to the heat conduction in ScYCBr_2_ and Sc_2_CBr_2_ being the role of other mechanisms negligible. This conclusion is compatible with results reported in other MXenes.^66^ Therefore, for the rest of the discussion, we will focus only on the three phonon scattering processes.

**Figure 7:**
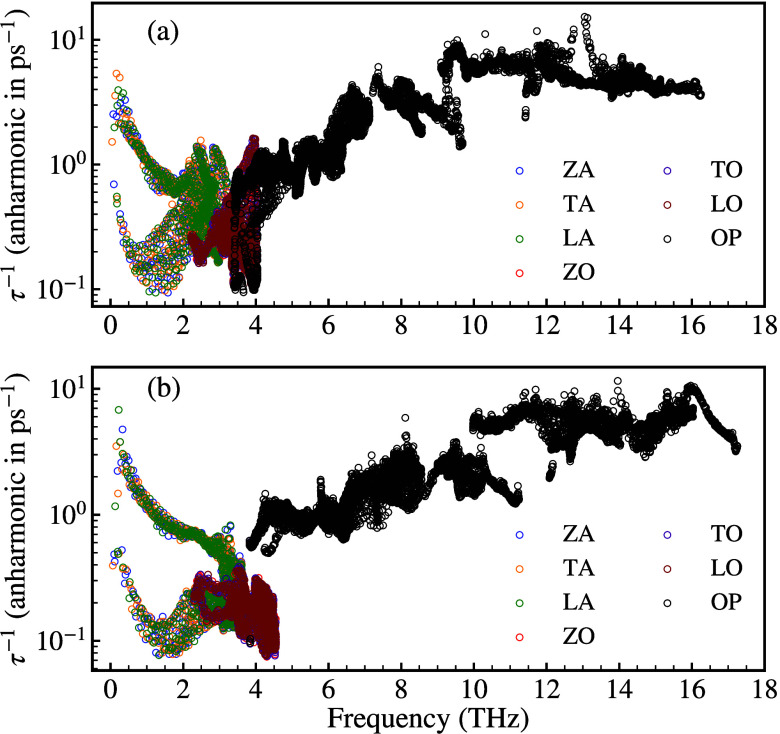
Anharmonic three-phonon scattering rates of the (a) Janus-like ScYCBr_2_ and (b) symmetric Sc_2_CBr_2_ MXene. The phonon–phonon scattering rates are calculated on a 100 × 100 × 1 **q**-grid mesh. The plot also shows the contribution of each phonon mode.

The scattering rates at 300 K for the anharmonic three-phonon scattering interactions are displayed in Figure 7. In both compounds, ScYCBr_2_ and Sc_2_CBr_2_, the relaxation time of the acoustic modes is shorter than those of the optical modes. In addition, the relaxation time of the acoustic modes is the shortest in ScYCBr_2_. The acoustic phonon relaxation time is concentrated between 10^–1^ ps and 10° ps, while the optical phonon relaxation time falls within the range of 10^–1^ ps and 10^+1^ ps. The longer phonon relaxation time of the acoustic modes is in accordance with their dominant contribution of the acoustic branches to κ_*L*_ (see Figure S9 in the Supporting Information). Moreover, further comparison indicates that most of the acoustic LA, and optical ZO and LO modes have a much stronger phonon scattering in the asymmetric MXene than in the symmetric one. It is also noted that for the Janus structure, we have higher phonon scattering rates, which implies that more modes are active at a given temperature. Thus, based on the lattice thermal conductivity eq 2, the value of the lattice thermal conductivity is mainly determined by the sum of the anharmonic three-phonon scattering and isotopic scattering rates (see Figure S8 in the Supporting Information), and the group velocity of phonons. This explains why the lattice thermal conductivity is slightly lower in the Janus-like ScYCBr_2_ than in the symmetric Sc_2_CBr_2_ MXene.

**Figure 8:**
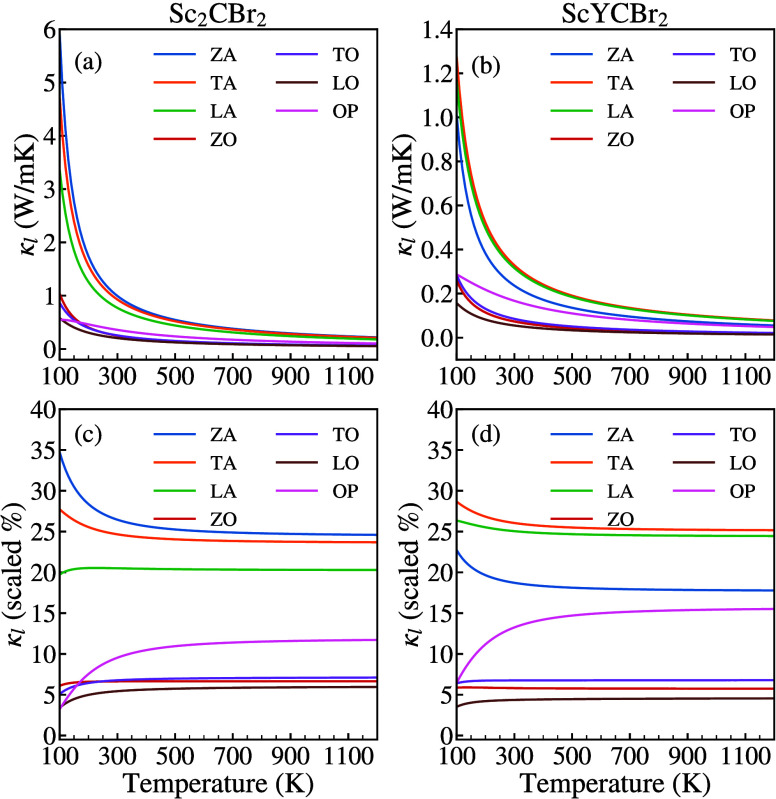
Temperature dependence of the thermal conductivity of each phonon mode chanel in κ_*L*_ of (a) symmetric Sc_2_CBr_2_ and (b) Janus-like ScYCBr_2_ MXene. Percentage contribution of each phonon mode to κ_*L*_ for of (c) symmetric Sc_2_CBr_2_ and (d) Janus-like ScYCBr_2_ MXene.

To understand how phonon transport works at its fundamental level in Sc_2_CBr_2_ and ScYCBr_2_, we have calculated the contributions of various phonon branches to the lattice thermal conductivity (See Figures 8a and 8b). Certain differences are evident for both materials. The contribution of the TA, LA, and ZA modes in the Janus-type structure is respectively around 29%, 22%, and 1% at temperatures ranging from 100 to 400 K and diminished by only 1% from 400 to 1200 K, see Figure 8(c). In contrast, in the asymmetric MXene material, the most involved channels are ZA, TA, and then LA, with a percentage of, respectively, 35%, 28%, and 20%, see Figure 8(d). Furthermore, we have observed that a channel from the optical branches somewhat contributes to the conductivity of the Janus-type structure. This is a consequence of the symmetry breaking in ScYCBr_2_. Figures 8a and 8b also illustrate how κ_*L*_ changes with temperature for each mode. These figures also show the evolution of κ_*L*_ with temperature. It is possible to attribute the difference in contribution between the asymmetric and symmetric structures to the different symmetry and, consequently, to the different selection rules and eigenvectors of the phonon modes.

**Figure 9:**
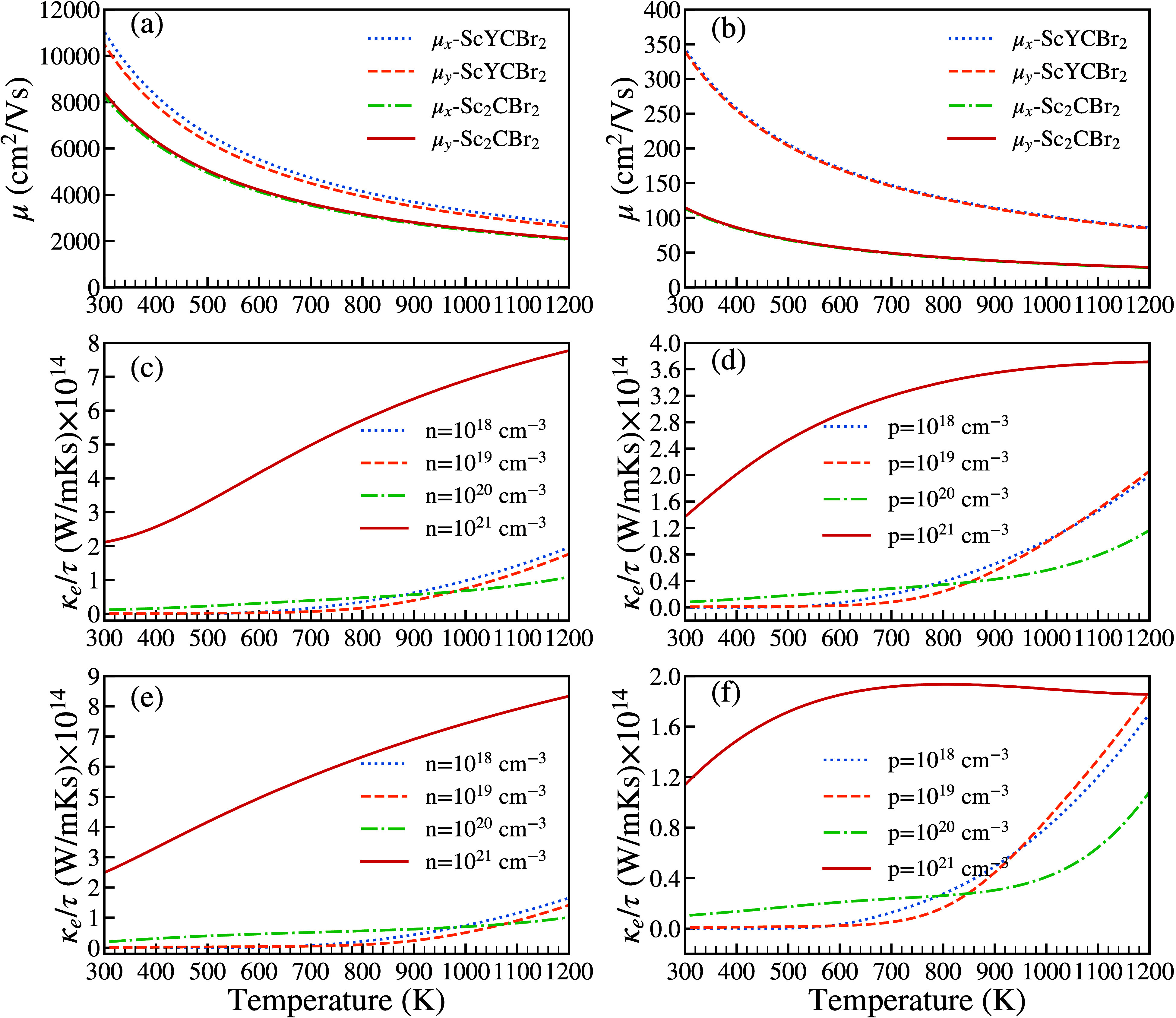
Carrier mobility μ of ScYCBr_2_ and Sc_2_CBr_2_. (a) gives the mobility of electrons and (b) the mobility of holes. The evolution of the electronic thermal conductivity (κ_*e*_/τ) as a function of temperature for (c) electrons and (d) holes, for different carrier charge concentrations of the Janus-type ScYCBr_2_ MXene. The evolution of the electronic thermal conductivity (κ_*e*_/τ) as a function of temperature for (e) electrons and (f) holes, for different carrier charge concentrations of the symmetric Sc_2_CBr_2_ MXene.

Once the most favorable material for thermoelectric applications and the most favorable scattering phonon channels are identified, it is necessary to check other TE properties. The thermoelectric efficiency is primarily associated with the value of the figure-of-merit (*ZT* = *S*^2^*σT*/(κ_*L*_ + κ_*e*_)), which depends also on the electronic thermal conductivity (κ_*e*_) and the conductivity (σ), being this proportional to the carrier mobility (μ). Indeed, because the mobility affects the capacity of electrons and holes to transport heat and electricity, the carrier mobility is also a crucial factor in the study of thermoelectric materials. In Figure 9, we present results of the carrier mobility for ScYCBr_2_ and Sc_2_CBr_2_. They have been calculated for both types of charge carriers using the deformation potential theory.^67^ This theory has been shown to be a reliable method for predicting the carrier mobility of 2D semiconductors, including MoS_2_. A detailed description of the methodology is given in the Supporting Information.

The values of the electron and hole mobilities at room temperature are given in Table S2 of the Supporting Information. The values have been extracted from the applications of strains on the band structure plots. See Figures S10, S11, and S12 in the Supporting Information. As the mobility of the charge carriers is related to the scattering rate and dispersion of bands in the band structures, we have taken care to use the band structures calculated using the HSE06 functional,^36^ one of the most accurate descriptions for such calculations. According to our results, the electron mobility is larger than the hole mobility. This is a consequence of the fact that near the Fermi level, the valence band (see Figure S13 in the Supporting Information) is less dispersive than the conduction band. The flat valence band leads to a large effective mass for the holes and consequently to a low mobility for them. At high temperatures, μ decreases for both types of carriers (see Figures 9(a) and 9(b)). We have also found that the electron and hole mobilities are different in the *x* and *y* transport directions, indicating that the carrier mobilities are anisotropic. Comparing the values of the mobility reported in the literature for similar structures, the anisotropy of the mobility in the studied compound is moderate.

In order to further study the electronic transport properties, we have used the Boltzrap2 code^42^ and we have considered a temperature-dependent relaxation times approximation as proposed by Casu et al.^68^ (see in the Supporting Information, the results in Figure S14, the global relaxation time values in Table S3, and the explanation of the method utilized to determine the relaxation time as a function of temperature). As mentioned before, the high performance of Janus-type ScYCBr_2_ MXene could be achieved thanks to its mechanical properties, its high electrical conductivity, its low thermal conductivity, and the potential of the material to generate electric power at a given temperature. Figure 9c–f illustrate how the electronic thermal conductivity increases drastically with temperature. This increase is clearly due to the change of the charge carrier concentration from 10^18^ cm^–3^ to 10^21^ cm^–3^ as well as to the enhancement of the electron group velocity at high temperatures. The plots also show that **n**-type carriers (i.e., electrons in the conduction band) are largely responsible for the electronic transport. In this case, the electronic thermal conductivity (κ_*e*_) is nearly four times larger in the case of electrons than in the case of holes. Such a characteristic made ScYCBr_2_ quite efficient for thermoelectric devices utilizing high-mobility carriers. Interestingly κ_*e*_ increases with temperature to attain 8 × 10^4^ W/(mK) at 1200 K. We have also found that κ_*e*_ is similar for BrSc_2_CBr_2_ and ScYCBr_2_. However, there are some differences, in the dependence of this magnitude on the hole charge carrier concentration (see for comparison Figure 9(c)–(f)). Nonetheless, compared to metallic Au, Ag, and Cu,^69^ the magnitude of the electronic thermal conductivity magnitude is still relatively moderate in the two studied materials. This is mostly due to the semiconducting nature of the studied material and the strong electron–phonon interaction in them, which produces comparatively short electron relaxation times.

**Figure 10:**
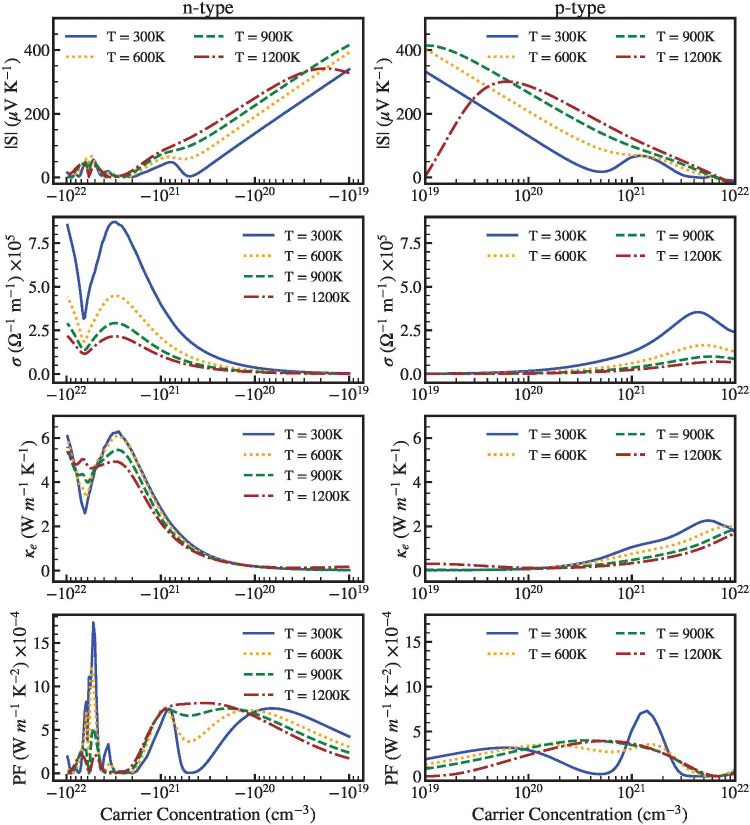
From the top to the bottom we present the evolution of the Seebeck coefficient, the electrical conductivity, the electronic thermal conductivity, and the panel power factor, as a function of **n**-type (left) and **p**-type (right) carrier concentration of ScYCBr_2_. The TE properties decrease with an increase in temperature because τ increases with it, see Table S3 in the Supporting Information.

**Figure 11:**
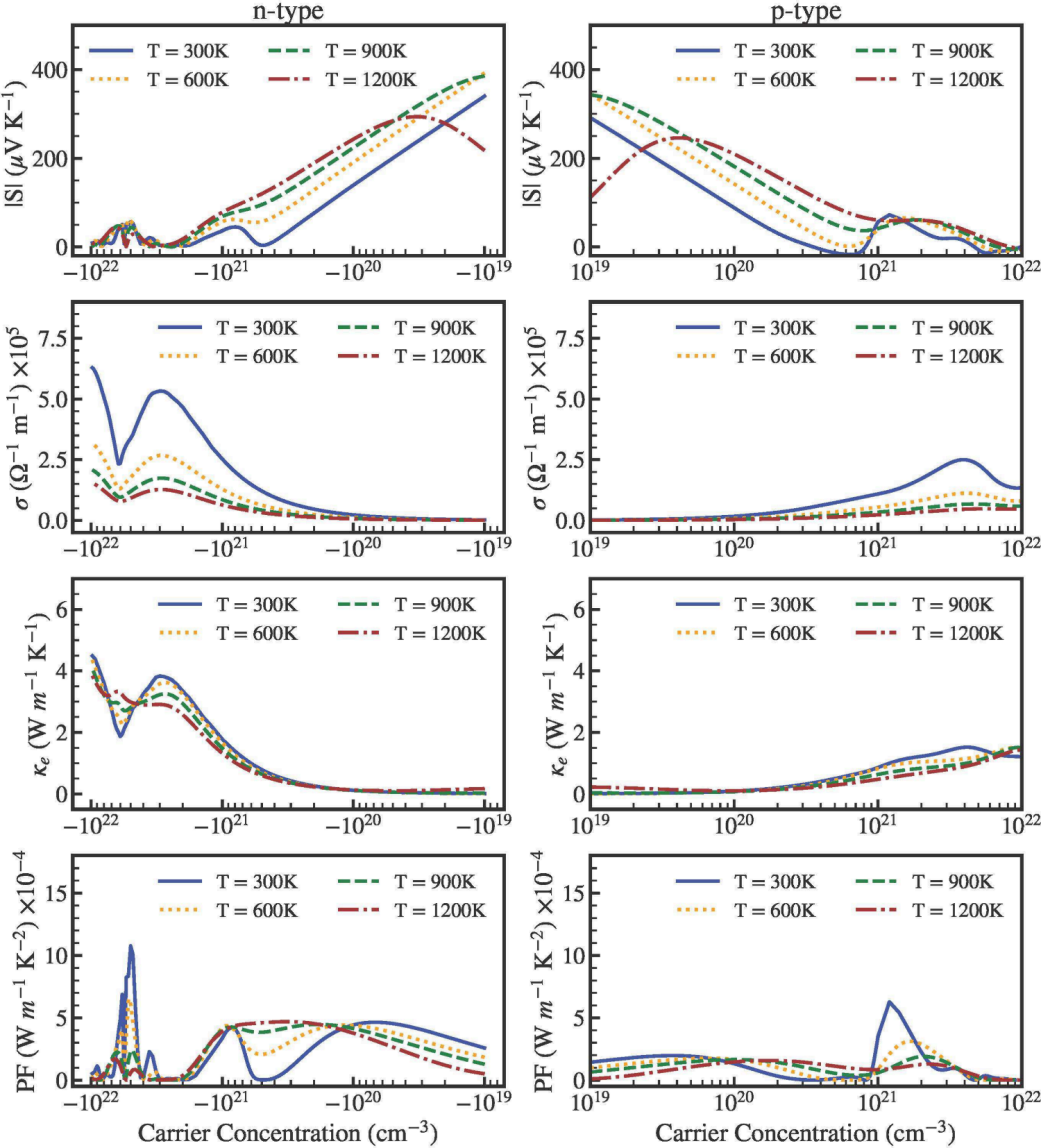
From the top to the bottom we present the evolution of the Seebeck coefficient, the electrical conductivity, the electronic thermal conductivity, and the panel power factor, as a function of **n**-type (left) and **p**-type (right) carrier concentration of Sc_2_CBr_2_. The TE properties decrease with an increase in temperature because τ increases with it, see Table S3 in the Supporting Information.

In order to gain a more thorough comprehension of our findings we have also studied the thermoelectric coefficients as a function of carrier concentration at 300, 600, 900, and 1200 K. Figures 10 and 11 display the electrical conductivity, σ, power factor, *PF* = *S*^2^σ, the absolute value of the Seebeck coefficient |*S*|, and the electronic thermal conductivity, κ_*e*_. To understand the behavior of these properties from Figures 10 and 11, we should remember that in our approximation the relaxation is a function of temperature.^68^ The *S* value of **n**-type carriers is close to the *S* value of **p**-type carrier. Comparing the two compounds ScYCBr_2_ is the material with the highest *S*. In spite of this, both structures can achieve *S* = 300 μVK^–1^ at 1200 K for both types of carriers at a moderate carrier concentration (10^20^ cm^–3^). On the other band, the low *S*-coefficient (brown dashed lines) found for both charge types at 1200 K with the carrier concentration beyond ×10^21^ cm^–3^ can be attributed to a bipolar conduction effect. This is a common effect for narrow gap semiconductors, like the materials here studied with band gap energies ∼1.5 eV. This effect makes the absolute value of the Seebeck coefficient to increase initially and then decrease at high temperatures.^70^

As it can be also discerned from Figures 10 and 11, our computed electrical transport coefficient and σ follow the expected trend for both types of carrier concentrations. Here, because there are more charge carriers, the electrical conductivity rises with carrier concentration following the Mott equation. The more dispersive character of the bands in the conduction region may be the reason for the higher electrical conductivity values observed for **n**-type carrier concentrations. When the concentration of **n**-type carriers is equal to 10^21^ cm^–3^, we obtain higher power factor values at 300 K of ∼8 × 10^21^ Wm^–1^K^–2^s^–1^ (for a time relaxation τ = 1.13 × 10^–14^s), even though the values of **p**-type carriers are nearly two times smaller. This high value can be attributed to the unusually high electrical conductivity. In terms of electronic thermal conductivity, it follows the same pattern as the electrical conductivity. For high temperatures, we can see that the electrical conductivity rises to reach a value of 4 × 10^21^ Wm^–1^K^–2^s^–1^. In the case of Sc_2_CBr_2_, σ reaches relatively smaller values: ∼ 8 × 10^21^ Wm^–1^K^–2^s^–1^ for the **n**-type and ∼2 × 10^21^ Wm^–1^K^–2^s^–1^ for the **p**-type charges.

The power factor is a relevant parameter for characterizing the electronic transport properties. It is dependent on both *S* and σ. *S* and it is inversely proportional to the carrier concentration, whereas σ increases as the carrier concentration rises. Because of the larger electrical conductivity, for **n**-type carriers the *PF* in both materials is always higher than the *PF* for **p**-type carriers. In addition, due to the inverse relationship between the electrical conductivity and temperature, the *PF* value falls with increasing temperature. More significantly, we can observe multiple peaks, with the most important occurring at a concentration of 10^21^ cm^–3^. These results suggest that heat can be efficiently converted into electrical power using the Janus structure.

**Figure 12:**
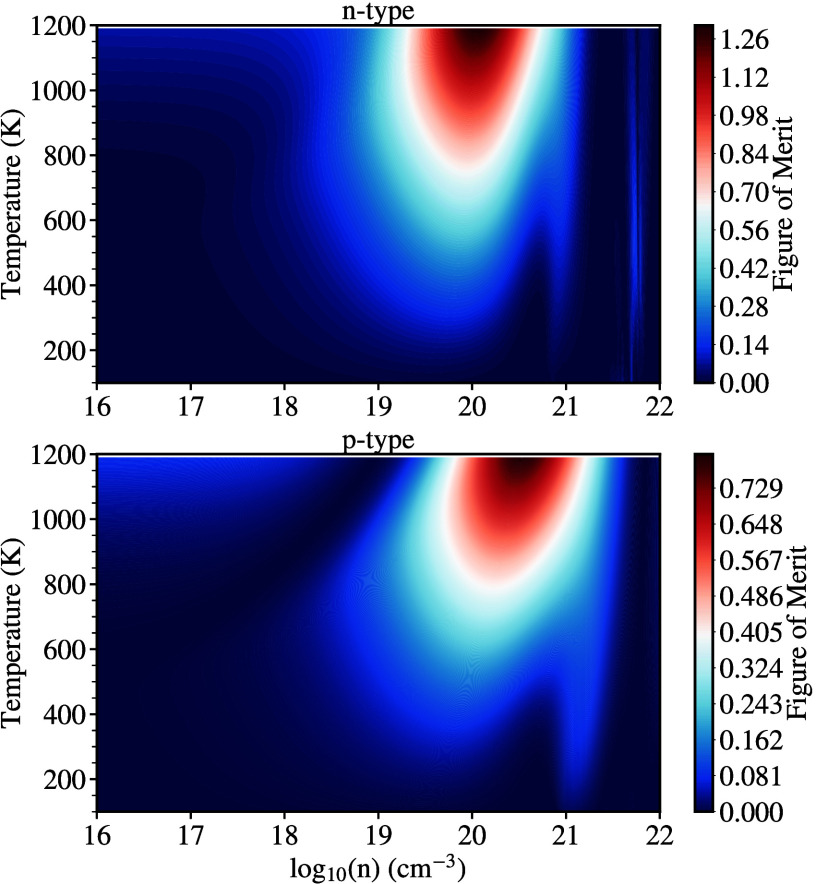
Figure of merit (*ZT*) map as a function of temperature and carrier charge concentration for both **n**-type and **p**-type carriers for ScYCBr_2_.

**Figure 13:**
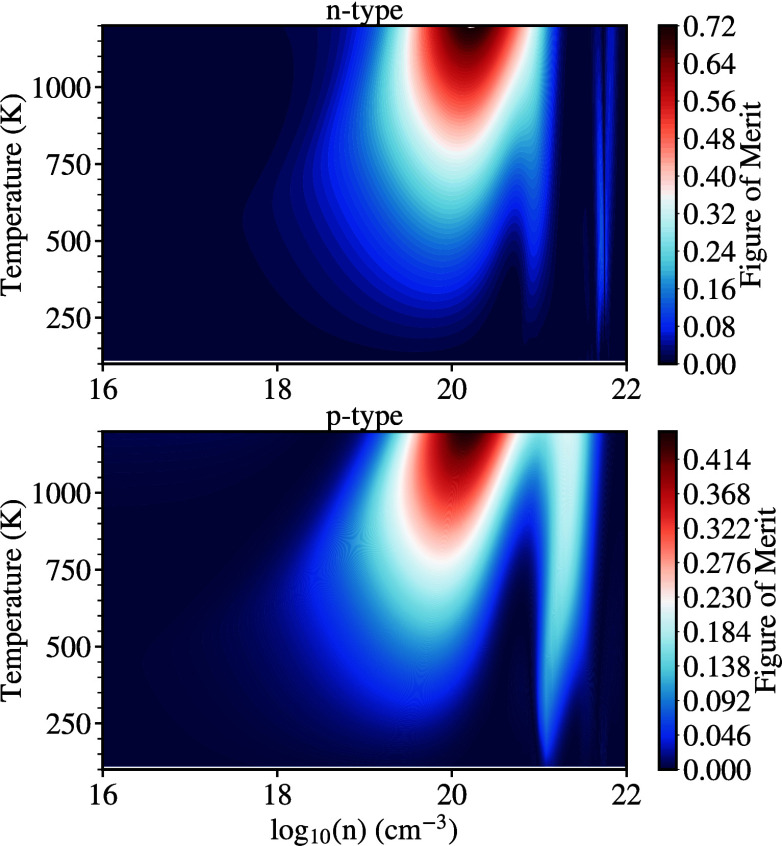
Figure of merit (*ZT*) map as a function of temperature and carrier charge concentration for both **n**-type and **p**-type carriers for Sc_2_CBr_2_.

Finally, we will examine the thermoelectric performance of both ScYCBr_2_ and Sc_2_CBr_2_ by evaluating the figure of merit (*ZT*). We start by outlining the functional relationship between *ZT* for different carriers and the carrier concentration vs various temperatures. The results are shown in the 2D maps of Figures 12, and 13. It is clear that at high temperatures, the optimal *ZT* is achieved for **n**-type carrier concentrations of ∼10^20^ cm^–3^ for both materials. However, the **p**-type carriers maximize the electrical transport but only at a large carrier concentration of 10^21^ cm^–3^. Such higher carrier concentration could be associated with an increased thermal conductivity, which can be detrimental to thermoelectric performance. We note that electrons are the main transport carriers for the investigated MXenes. Thus, with this behavior combined with the low thermal conductivity and the dynamical and thermal stability of the structures, we can conclude that Janus-like ScYCBr_2_ MXene meets the requirements for being a high-efficiency thermoelectric material. The *ZT* value reached respective values of 1.2 and 0.7 for the **n**-type ScYCBr_2_ and Sc_2_CBr_2_, and 0.73 and 0.41 for the **p**-type materials. Such results are rather similar to those found by Murari et al. for similar structures.^64^

## Conclusions

Along this contribution, we have provided conclusive evidence that the asymmetric Janus-type ScYCBr_2_ MXene has very promising thermoelectric properties. We have achieved this conclusion from computational simulations of both phonon and electronic transport properties. The study is based on the density-functional theory, and it is complemented by calculations applying the phonon Boltzmann transport theory. We have found that 2D ScYCBr_2_ is dynamically and thermally stable from 0 K to high temperatures. Results have also shown that there is a significant degeneracy between the ZA and LA modes near the Γ – *M* point. The acoustic branches show a robust coupling between them, resulting in an important anharmonicity and low-mean-free paths of phonons. The properties of the related symmetric Sc_2_CBr_2_ MXene have also been studied and compared to the asymmetric ScYCBr_2_. Our findings suggest that, compared to Sc_2_CBr_2_, in ScYCBr_2_ the phonon anharmonicity is less relevant. We have also shown that the symmetry breaking in ScYCBr_2_ enhances the phonon scattering channels by making additional scattering centers for phonons and, consequently, reduces thermal transport. We have also found that the behavior of the lattice thermal conductivity κ_*L*_ is correlated with the phonon group velocity and phonon lifetime. Additionally, by assuming that the compounds could be doped with **n**-type and **p**-type impurities,^10^ we have demonstrated that the Janus-like ScYCBr_2_ MXene exhibits excellent **n**-type transport properties and high thermoelectric properties at both ambient temperature and 1200 K. In summary, this work has allowed us to understand the potential of ScYCBr_2_ as an excellent thermoelectric material. We think that the synthesis of this material for high-performance thermoelectric applications would be encouraged by our study.

## Data Availability

All relevant data are available from the corresponding author upon reasonable request.

## Supporting Information Available

Structural model of the studied materials, the methods used to estimate the carrier mobility, the anharmonic phonon dispersion plots, the lattice thermal conductivity, and the temperature-dependent relaxation time; several figures showing the third-order force constant interaction cutoff convergence tests, the molecular-dynamic simulation of temperature and total energy as a function of the time step, the specific heat capacity of both structures, the cumulative lattice thermal conductivity at 300 K, the three-phonon, four-phonon, and isotopic scattering rate as a function of frequency, and finally the band structure and relaxation time (PDF)
