# Supplementary material for: Janus ScYCBr2 MXene as a Promising Thermoelectric Material
Source: ACS Appl Energy Mater. 2024 Jul 22;7(15):6598–611. doi: 10.1021/acsaem.4c01221 (PMC11323026; doi:10.1021/acsaem.4c01221)
Supplement: Supplementary file 1 — ae4c01221_si_001.pdf [file ae4c01221_si_001.pdf]

Supporting Information
Janus $ScYCBr_2$ MXene as a Promising Thermoelectric Material

Mounir Ould-Mohamed,[*,†] Tarik Ouahrani,[*,‡,¶,§] Reda Boufatah,[‖] Ángel Morales-García,[⊥] Ruth Franco,[#] Michael Badawi,[§] and Daniel Errandonea[*,@]

†LPTHIRM, Département de Physique, Faculté des Sciences, Université Saâd Dahlab-Blida 1, B.P. 270 Route de Soumâa, 09000 Blida, Algeria

‡Ecole Supérieure en Sciences Appliquées, ESSA-Tlemcen, BB 165 RP Bel Horizon, Tlemcen 13000, Algeria.

¶Laboratoire de Physique Théorique, Université de Tlemcen, Tlemcen 13000, Algeria.

§Université de Lorraine, Laboratoire Lorrain de Chimie Moléculaire, CNRS, L2CM, F-57000 Metz, France

‖Laboratoire de Physique Théorique, Université de Tlemcen, Algeria.

⊥Departament de Ciéncia de Materials i Química Física and Institut de Química Teórica i Computacional (IQTCUB), Universitat de Barcelona, c/Martí i Franquès 1-11, 08028 Barcelona, Spain

#MALTA Consolider Team and Departamento de Química Física y Analítica, Universidad de Oviedo, E-33006 Oviedo, Spain.

@Departamento de Física Aplicada - Instituto de Ciencia de Materiales, Matter at High Pressure (MALTA) Consolider Team, Universidad de Valencia, Edificio de Investigación, C/Dr. Moliner 50, Burjassot, 46100, Valencia, Spain

E-mail: ouldmohamed_mounir@univ-blida.dz; tarik.ouahrani@univ-tlemcen.dz; daniel.errandonea@uv.es

Structural model of the studied materials

MXenes are the two-dimensional layered equivalents of the three-dimensional MAX compounds. They are produced by exfoliating the layers of group A elements linking the carbon/nitrogen X and transition metal M layers in MAX compounds. Different functional groups are used to passivate the dangling bonds on the surfaces during this process, resulting in MXenes with the chemical formula $M_{n+1}X_nT_x$ (n=1-3), where M, X, and T stand for the surface functional groups, carbon/nitrogen, and transition metal, respectively.[1] Naturally, MXenes have more compositional flexibility and functional property tunability than other 2D materials. In this work, we study MXene compounds built from the MXene with the chemical formula Sc_2C which has a hexagonal symmetry described by the space group $P6_3/mmc$ (No. 194), see Figure S1. Upon passivation of both surfaces with the functional group Br, Sc_2CBr_2 is obtained with a reduced trigonal symmetry described by space group $P\bar{3}m1$ (No. 164). It is also possible to build an asymmetric MXene by replacing one of the Sc layers with a Y layer, making $ScYCBr_2$, breaking the inversion symmetry, and creating a crystal structure described by space group $P3m1$ (No. 156). The last two 2D MXenes, Sc_2CBr_2 and $ScYCBr_2$ are the materials modeled in this work.

Elastic properties

For the 2D trigonal structure of the studied compounds, there are only two independent elastic constants in the elastic tensor, C_{11} and C_{12}, From symmetry considerations $C_{66} = \frac{C_{11}-C_{12}}{2}$. The strain energy curves corresponding to uniaxial and biaxial tensile strains have been fitted within the linear response region to determine the elastic constants of $ScYCBr_2$ and Sc_2CBr_2 using strains with an increment of 0.25%. The calculated elastic constants are summarized in Table S1. Their values fulfill the Born-Huang stability criteria[2,3] ($C_{11} > 0$ and $C_{66} > 0$) supporting the mechanical stability of both materials. We found that C_{11} is significantly larger than C_{12}, i.e. axial compression requires larger stresses than shear and

tensile deformations. Using the elastic constants, we derived the in-plane Young's modulus ($Y_{2D} = \frac{C_{11}^2 - C_{12}^2}{C_{11}}$), shear modulus ($G = C_{66}$), and Poisson ratio ($\nu = \frac{C_{12}}{C_{11}}$)[4] of $ScYCBr_2$ and Sc_2CBr_2. The calculated elastic moduli are shown in Table S1. The in-plane Young's modulus (Y_{2D}) of $ScYCBr_2$ is comparable to that of the MoS_2 monolayer (130 $N.m^{-1}$)[5] and the PB monolayer (136 $N.m^{-1}$).[6] The Poisson ratio is similar to that of sedimentary rocks like limestone.

Calculation of anharmonic phonon dispersion and lattice thermal conductivity

The temperature-dependent phonon dispersions were obtained using a self-consistent phonon theory (SCPH). The self-consistent phonon mode computes temperature-dependent phonon frequencies by solving the following equation self-consistently:[7]

$$V_{\boldsymbol{q}ij}^{[1]} = \omega_{\boldsymbol{q}i}^2 \delta_{ij} + \frac{1}{2} \sum_{\boldsymbol{q}_1,k} F_{\boldsymbol{q}\boldsymbol{q}_1,ijkk} \frac{\hbar \left[1 + 2n(\omega_{\boldsymbol{q}_1 k})\right]}{2\omega_{\boldsymbol{q}_1 k}}. \tag{S1}$$

Here, $\omega_{\boldsymbol{q}i}$ is the harmonic phonon frequency and $F_{\boldsymbol{q}\boldsymbol{q}_1,ijkk}$ is the reciprocal representation of high-order force constants computed using the harmonic eigenvectors. This matrix is the computationally most expensive part of the calculations and requires convergence tests. We have considered up to the eighth-nearest neighbors (8-NN) for the calculations of the third-order force constants (IFCs), which leads respectively to 395 (841) configurations for Sc_2CBr_2 ($ScYCBr_2$) to be calculated. These configurations are constructed by displacing an atom from its equilibrium position by 0.01 Å. The second and third-order force constants can then be extracted by solving a least-square problem after obtaining the atomic forces from the density-functional theory (DFT) calculations for each configuration. At finite temperatures, we extract the force constants up to the fourth order by a state-of-the-art Compressing Sensor (CS) method.[8] The harmonic and anharmonic IFCs are used to compute the lattice thermal

conductivity κ_l and solve the Boltzmann transport equation for phonons. The convergence of κ_l for the used cutoff is presented in Figure S2.

Carrier mobility

The carrier mobility μ was calculated using the deformation potential theory developed by Bardeen and Shockley. Under this approximation μ can be expressed as:[9]

$$\mu = \frac{e\hbar^3 C_{2D}}{k_B T m^* m_d E_d^2} \tag{S2}$$

where e, $\hbar$, k_B, E_d, and T are the electron charge, the Planck constant, the Boltzmann constant, the deformation potential, and the temperature, respectively. On the other hand,m^* is the carrier effective mass along the transport direction. m^* can be expressed by:

$$\frac{1}{m^*} = \pm\frac{1}{\hbar^2}\left|\frac{\partial^2 E(k)}{\partial k^2}\right| \tag{S3}$$

where k and E(k) are the wave vector and the corresponding energy dispersion, respectively. $m_d = \sqrt{m_x m_y}$ is the average effective mass, where m_x and m_y are the effective mass along x and y directions, respectively. Finally, the elastic modulus C_{2D} can be obtained using the the following equation :

$$C_{2D} = \frac{1}{S}\frac{\partial^2 E}{\partial \epsilon_{uniaxial}^2} \tag{S4}$$

where E is the total energy of the system and S is the area of the supercell at equilibrium.

Temperature-dependent relaxation time

To build a model that describes the realistic operation of thermoelectric applications, based on the work of Jacoboni *et al.*,[10] we have estimated the relaxation time as a function of temperature. These calculations have been recently included in the BoltzTrap[11] framework by

the group of V. Fiorentini.[12] Based on established semi-classical theories, analytical energy-dependent expressions for the relaxation time were created. These expressions describe the principal mechanisms of electron scattering caused by charged impurities, polar-optical phonons, and acoustic phonons.

$$\tau_{ac}(E,T) = \frac{2\pi\hbar^4\rho v^2}{(2m^*)^{\frac{3}{2}} k_B T D_{ac}^2 \sqrt{E}}, \tag{S5}$$

where E is the electron energy and T is the temperature. All other parameters are defined in Table S4. We have modeled the optical phonon scattering with an elastic deformation potential, which is similar to the assumptions that were used in acoustic phonon scattering (D_{op}):

$$\tau_{op}(E,T) = \frac{\sqrt{2k_BT}\pi x_o \hbar^2 \rho}{m^{*\frac{3}{2}} D_{op}^2 [\ N_{op}\sqrt{x+x_o} + (N_{op}+1)\Theta(x-x_o)\sqrt{x-x_o}]}, \tag{S6}$$

$$N_{op} = \frac{1}{\exp \frac{\hbar\omega_{op}}{k_BT} - 1}, x = \frac{E}{k_BT}, x_o = \frac{\hbar\omega_{op}}{k_BT}. \tag{S7}$$

The absorption of optical phonons by electrons is represented by the first term in the denominator of Eq. S6, while the emission of optical phonons by electrons is represented by the second term. The Heaviside step function Θ included in the second term represents the probability of emission of a phonon when $E < \hbar\omega_{op}$, which is zero because the electron does not have enough energy to emit the phonon. The number of optical phonons is N_{op}.

The polar optical scattering is modeled following Ridley:[13]

$$\tau_{pop}(E,T) = \sum_i \frac{Z(E,T,\omega_i^l)E^{\frac{3}{2}}}{C(E,T,\omega_i^l) - A(E,T,\omega_i^l) - B(E,T,\omega_i^l)} \tag{S8}$$

where the sum is over all longitudinal-optical phonons, with energy $\omega_i^l\ i$; the functions A, B, C, and Z are omitted for brevity and can be found in Ref.[14]

For the impurity scattering, we use the Brooks-Herring approach:

$$\tau_{imp}(E,T) = \frac{E^{\frac{3}{2}}\sqrt{2m^*}4\pi\varepsilon^2}{(log(1+\frac{1}{x}) - \frac{1}{1+x})\pi n_I Z_I^2 e^4} \quad \text{with} \quad x = \frac{E}{k_B T}. \tag{S9}$$

Ultimately, a piezoelectric field is produced in semiconductors by the strain brought on by acoustic phonons. The model used to describe this piezoelectric scattering was obtained from Ref.[10]

$$\tau_{pac}(E,T) = \frac{\sqrt{2E}2\pi\varepsilon^2\hbar^2\rho v^2}{p^2 e^2\sqrt{m^*}k_B T} \times \left[1 - \frac{\epsilon_o}{2E}\log(1+4\frac{E}{\epsilon_o}) + \frac{1}{1+4\frac{E}{\epsilon_o}}\right] \tag{S10}$$

where $\varepsilon = \epsilon_o + \epsilon_\infty$ and the piezoelectric effect is captured by the piezoelectric constant, p.

The global relaxation time is then obtained using the rule of Matthiessen (for results see Figure S13):

$$\frac{1}{\tau_{total}(E,T)} = \frac{1}{\tau_{imp}(E,T)} + \frac{1}{\tau_{ac}(E,T)} + \frac{1}{\tau_{op}(E,T)} + \frac{1}{\tau_{pop}(E,T)} + \frac{1}{\tau_{pac}(E,T)}. \tag{S11}$$

Table S1 Calculated values of elastic constants C_{ij} (in $N.m^{-1}$), Young's modulus Y_{2D} (in $N.m^{-1}$), Poisson ratio (ν), and shear modulus (in $N.m^{-1}$), for $ScYCBr_2$ and Sc_2CBr_2.

	C_{11}	C_{12}	C_{66}	Y_{2D}	ν	G
$ScYCBr_2$	146.00(5)	42.31(5)	52.00(5)	134.40(5)	0.23(5)	52.00(5)
Sc_2CBr_2	160.78(5)	35.30(5)	62.74(5)	153.03(5)	0.22(5)	62.74(5)

Table S2 The carrier effective mass m^* (in units of the free electron mass m_0), elastic modulus C_{2D} (in Nm^{-1}), E_d (in eV), and the mobility of carriers μ (in $cm^2V^{-1}s^{-1}$) along the x and y direction of $ScYCBr_2$, and Sc_2CBr_2, monolayers at T = 300 K. All parameters used to calculate μ were extracted from Figures S10 and S11.

		m_x^*	m_y^*	C_{2D}^x	C_{2D}^y	E_{2D}^x	E_{2D}^y	μ_x	μ_y
$ScYCBr_2$	Electron	0.114(1)	0.397(1)	199.32(1)	196.99(1)	-3.450(1)	-3.520(1)	11042.7(1)	10483.9(1)
	Hole	0.291(1)	0.660(1)	199.32(1)	196.99(1)	-6.530(1)	-6.530(1)	343.5(1)	339.5(1)
Sc_2CBr_2	Electron	0.296(1)	0.303(1)	209.77(1)	211.38(1)	-3.520(1)	-3.500(1)	8256.5(1)	8415.2(1)
	Hole	0.220(1)	1.410(1)	209.77(1)	211.38(1)	-7.090(1)	-7.070(1)	113.3(1)	114.8(1)

Table S3 Calculated values of the total relaxation time ($\tau_{total}(E,T)$) as a function of the temperature for $ScYCBr_2$ and Sc_2CBr_2, all components are in units of 10^{-14}s

Temperature (K)	300	600	900	1200
$ScYCBr_2$	1.135(1)	0.597(1)	0.400(1)	0.308(1)
Sc_2CBr_2	0.767(1)	0.393(1)	0.264(1)	0.199(1)

Table S4 Symbols and units for the scattering parameters required in various scattering models.

Parameter	Symbol	Units
Mass density	ρ	kg/m^3
Lattice constant	a	m
Low freq. dielectric constant	ϵ_0	-
High freq. dielectric constant	ϵ_∞	-
Acoustic velocity	v	m/s
Effective mass ratio	m^*	-
Acoustic deformation potential	D_{ac}	eV
Optical deformation potential	D_{op}	eV
Optical phonon energy	$\hbar\omega_{op}$	eV
Number of impurities	n_I	cm^{-3}
Charge on impurity	Z_I	-
Piezoelectric constant	p	C/m^2

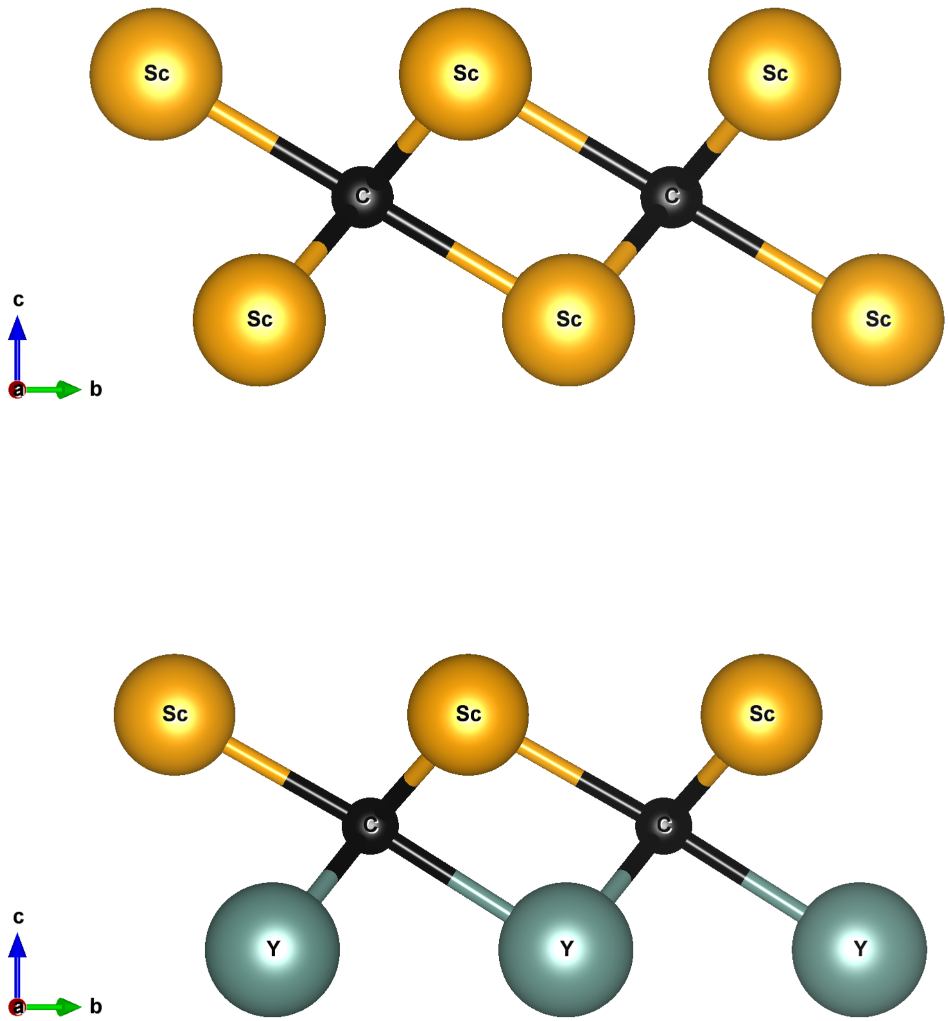

Figure S1 Schematic representation of the structure of Sc_2C and ScYC MXenes before passivation of surfaces with Br.

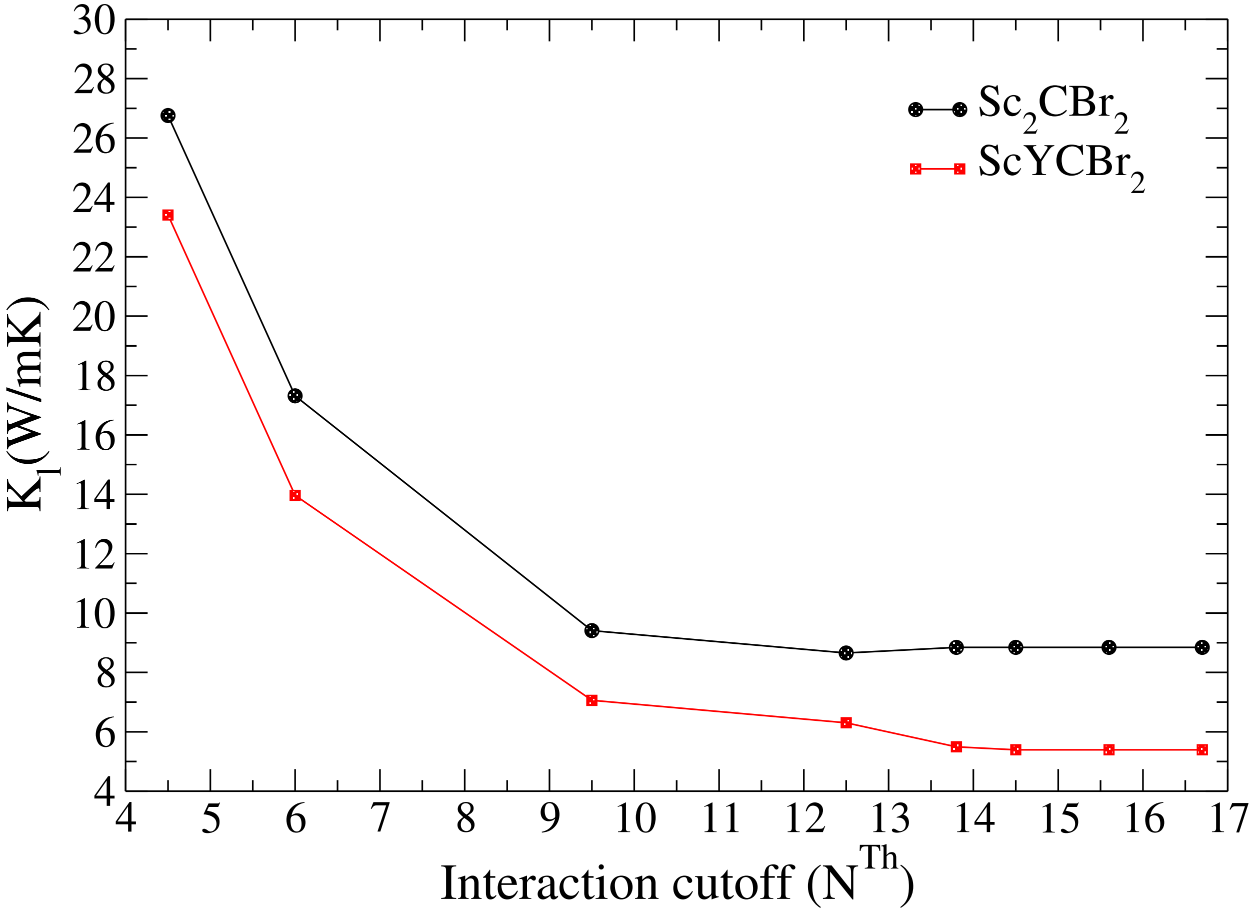

Figure S2 Third-order force constant interaction cutoff convergence tests for the thermal conductivity at 300 K for $ScYCBr_2$ and Sc_2CBr_2.

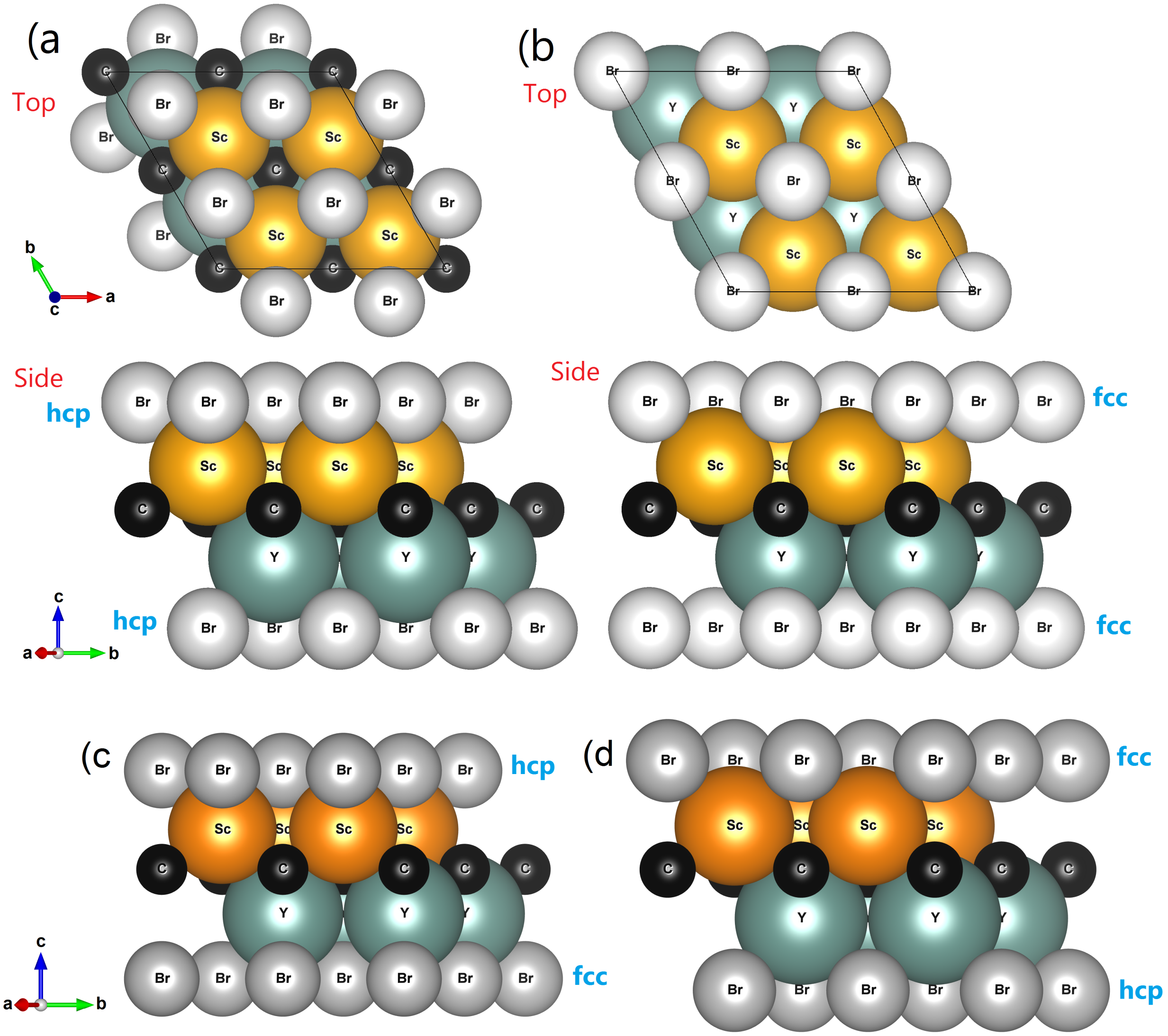

Figure S3 Schematic representation of the Janus $ScYCBr_2$ MXene: (a) and (b) show respectively the top and side view of the hcp-hcp and fcc-fcc hollow sites described in the text; (c) and (d) show the side view of the hcp-fcc and fcc-hcp hollow sites. Grey, dark yellow, black, and metallic green spheres represent Br, Sc, C, and Y atoms, respectively

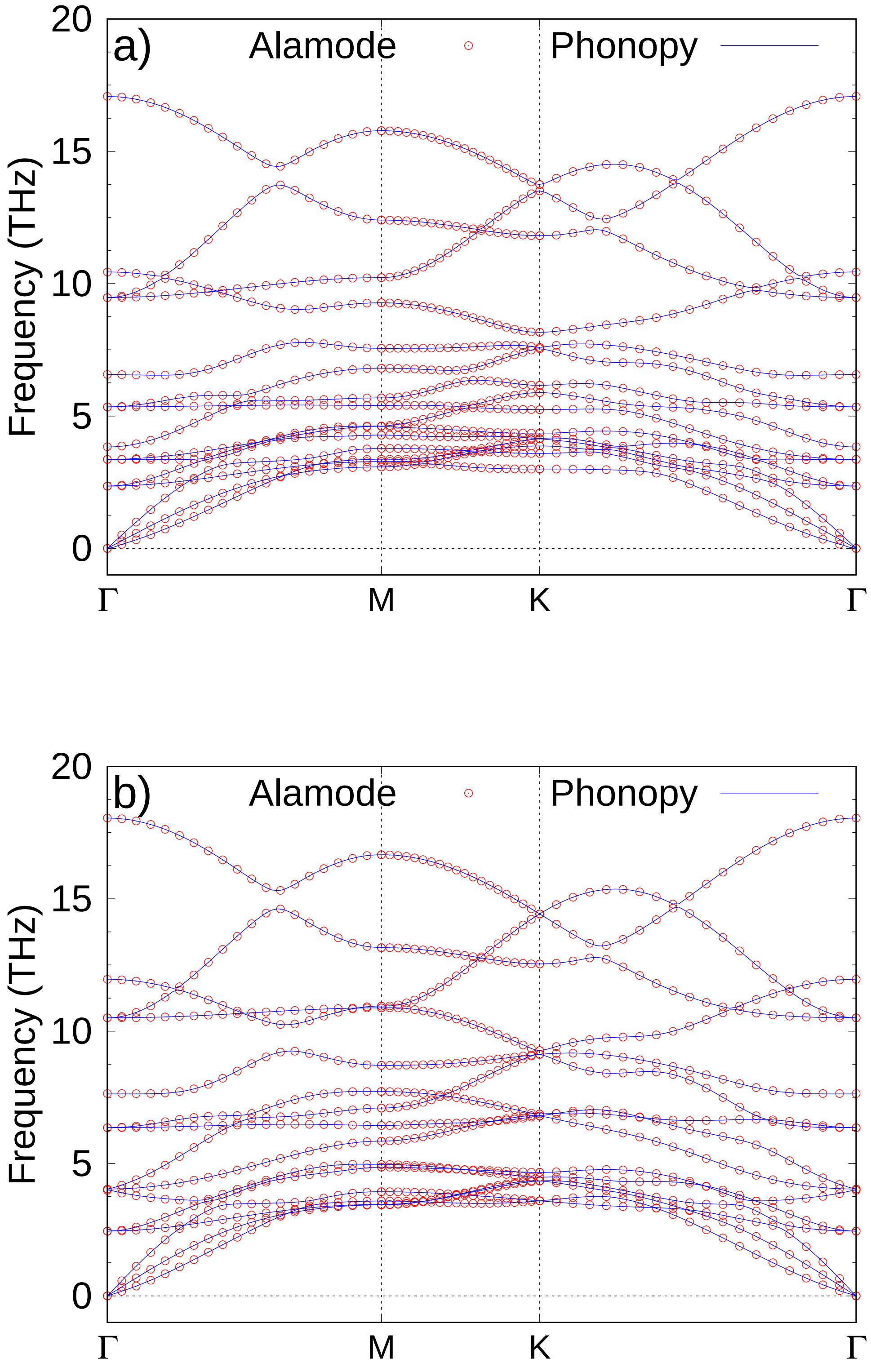

Figure S4 The phonon dispersion calculated at 0 K temperature for (a): $ScYCBr_2$, and (b): Sc_2CBr_2 The plot represents the results obtained using both the Phonopy (blue solid line) and Alamode (red circle) codes.

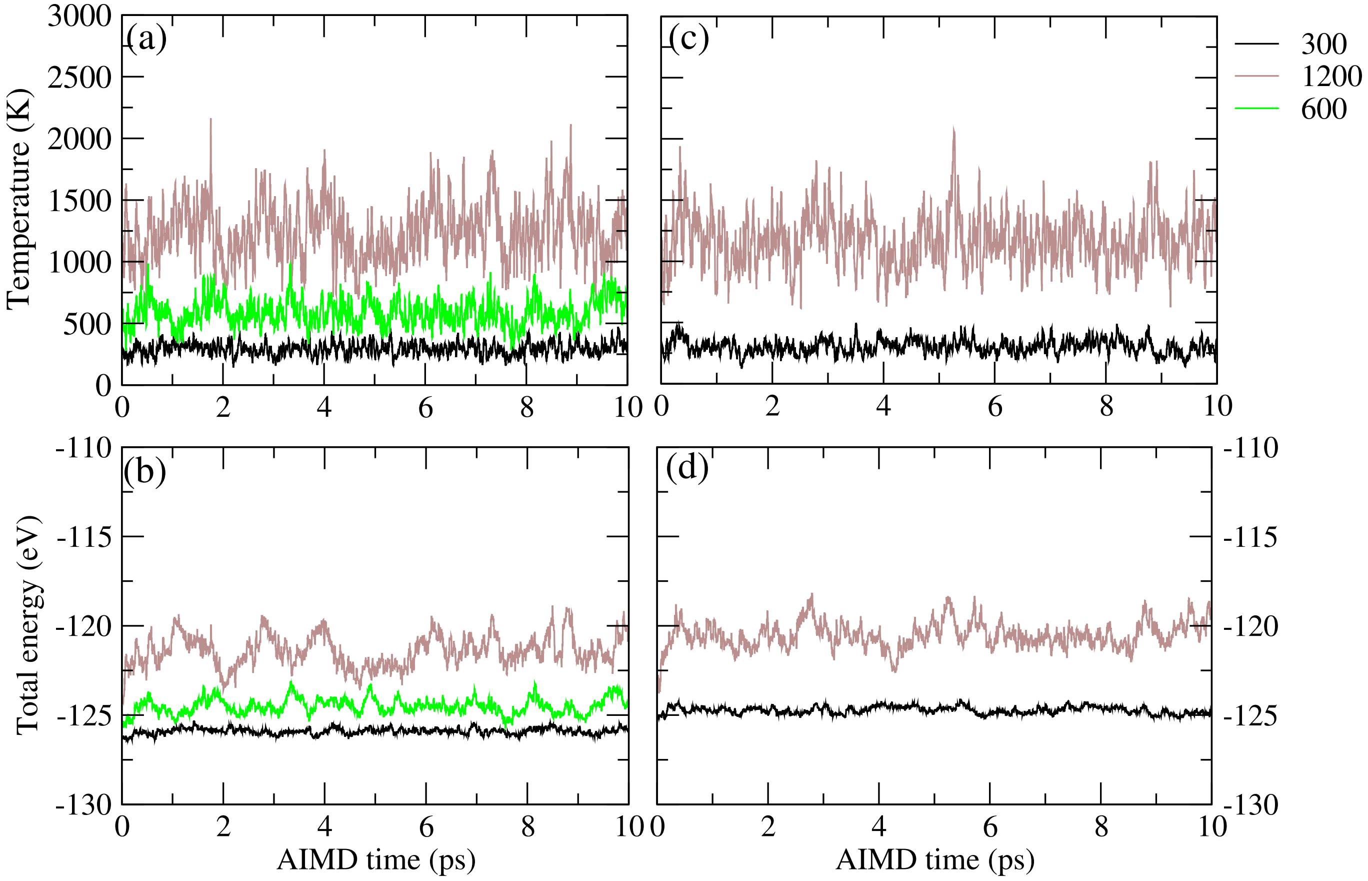

Figure S5 Molecular-dynamic simulation of temperature and total energy as a function of time step in (ps) for $ScYCBr_2$ in (a) and (b), and Sc_2CBr_2 in (c) and (d).

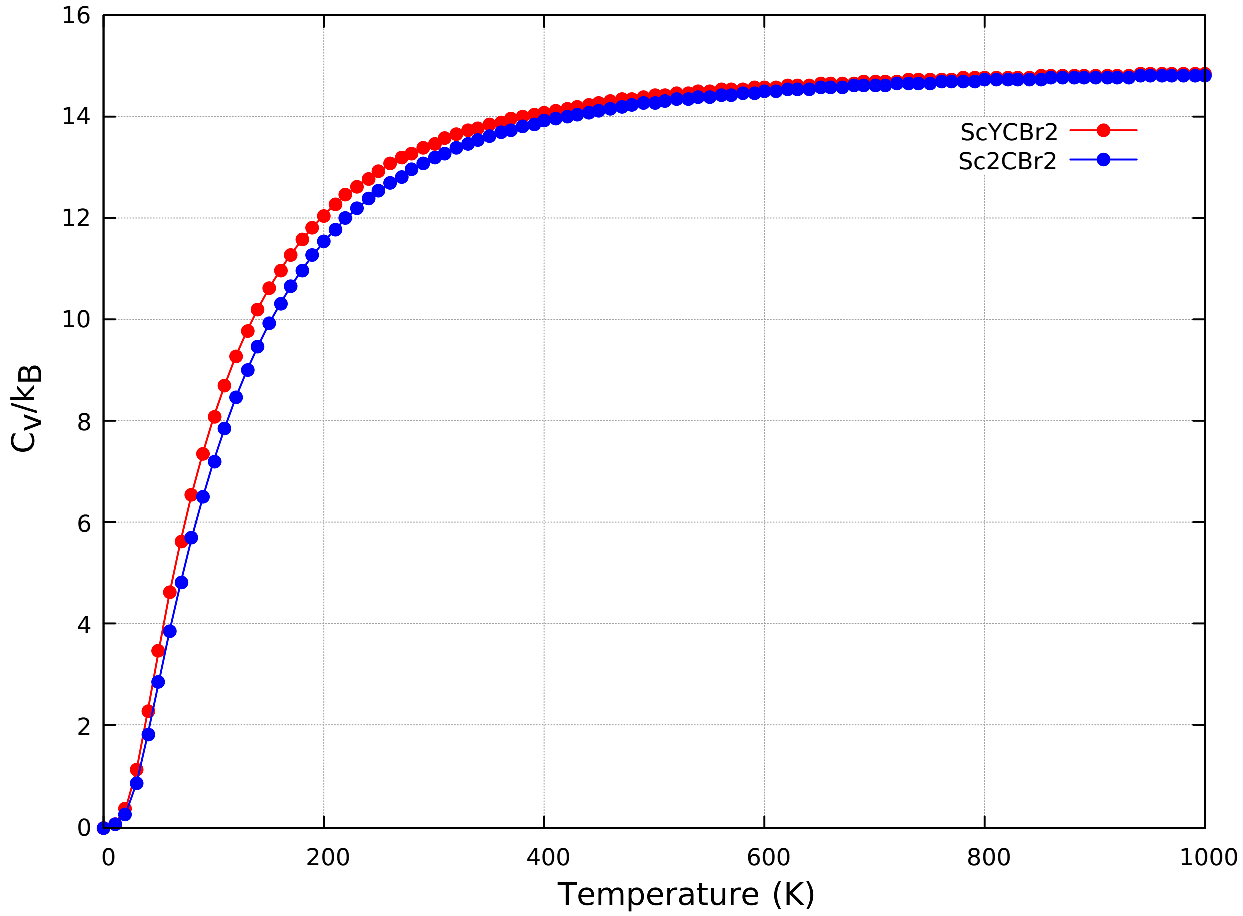

Figure S6 The specific heat capacity of Sc_2CBr_2 and $ScYCBr_2$ as a function of temperature. The plot is given in a unit of J/(mol. K).

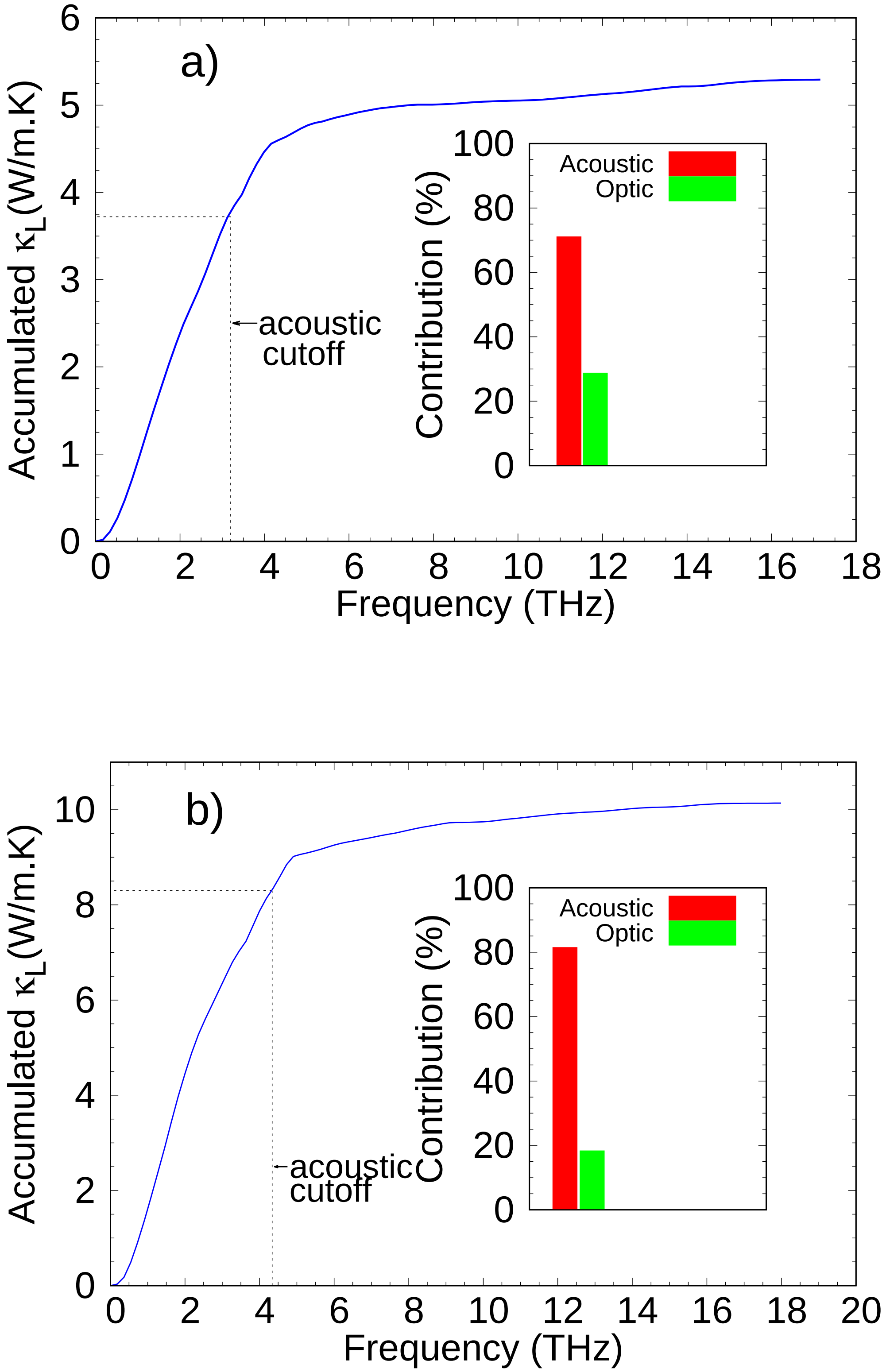

Figure S7 The cumulative lattice thermal conductivity at 300 K as a function of phonon frequency for (a) $ScYCBr_2$ and (b) Sc_2CBr_2.

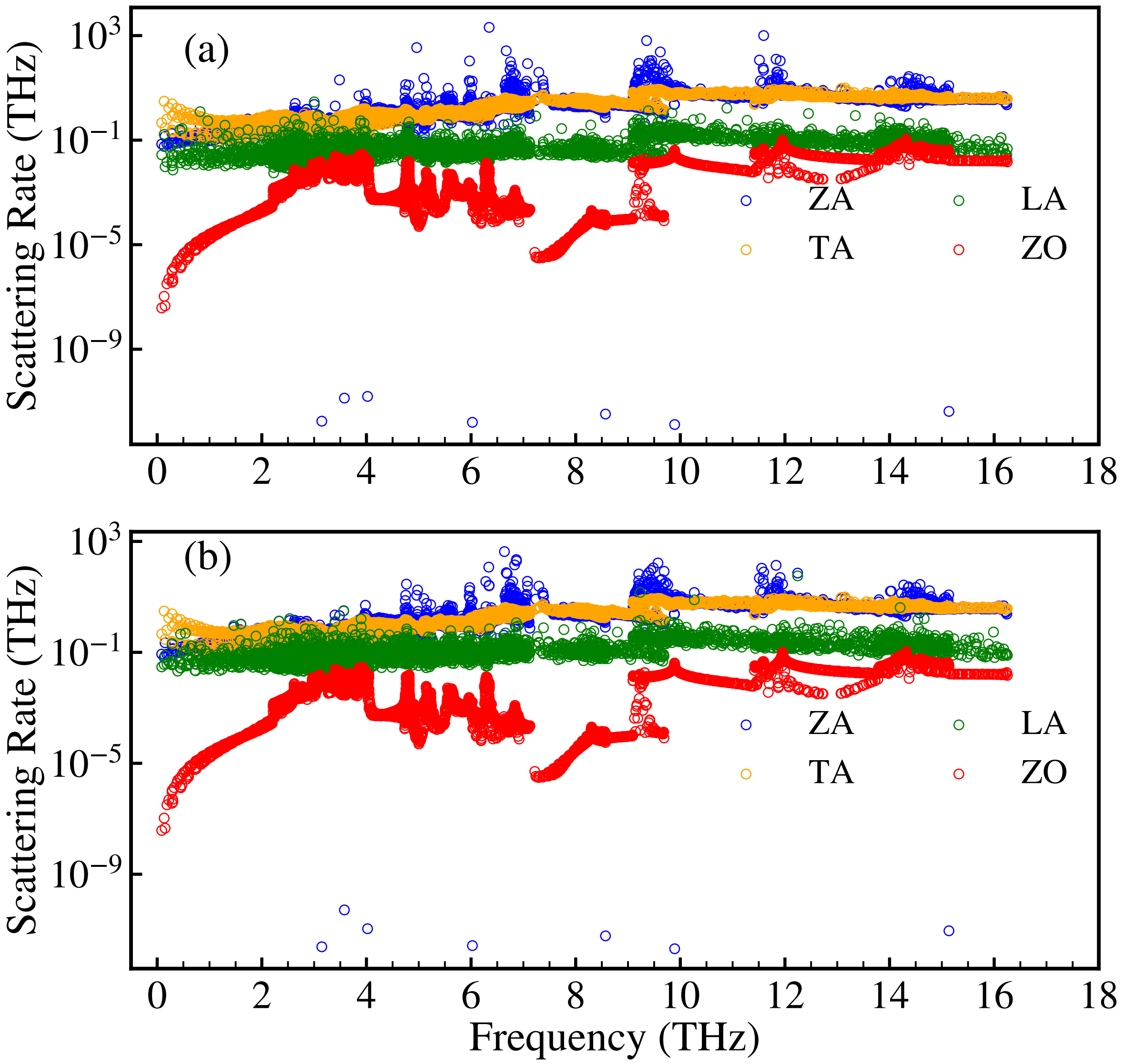

Figure S8 Three-phonon, four-phonon, and isotopic scattering rates functions of frequency for (a) $ScYCBr_2$ and (b) Sc_2CBr_2.

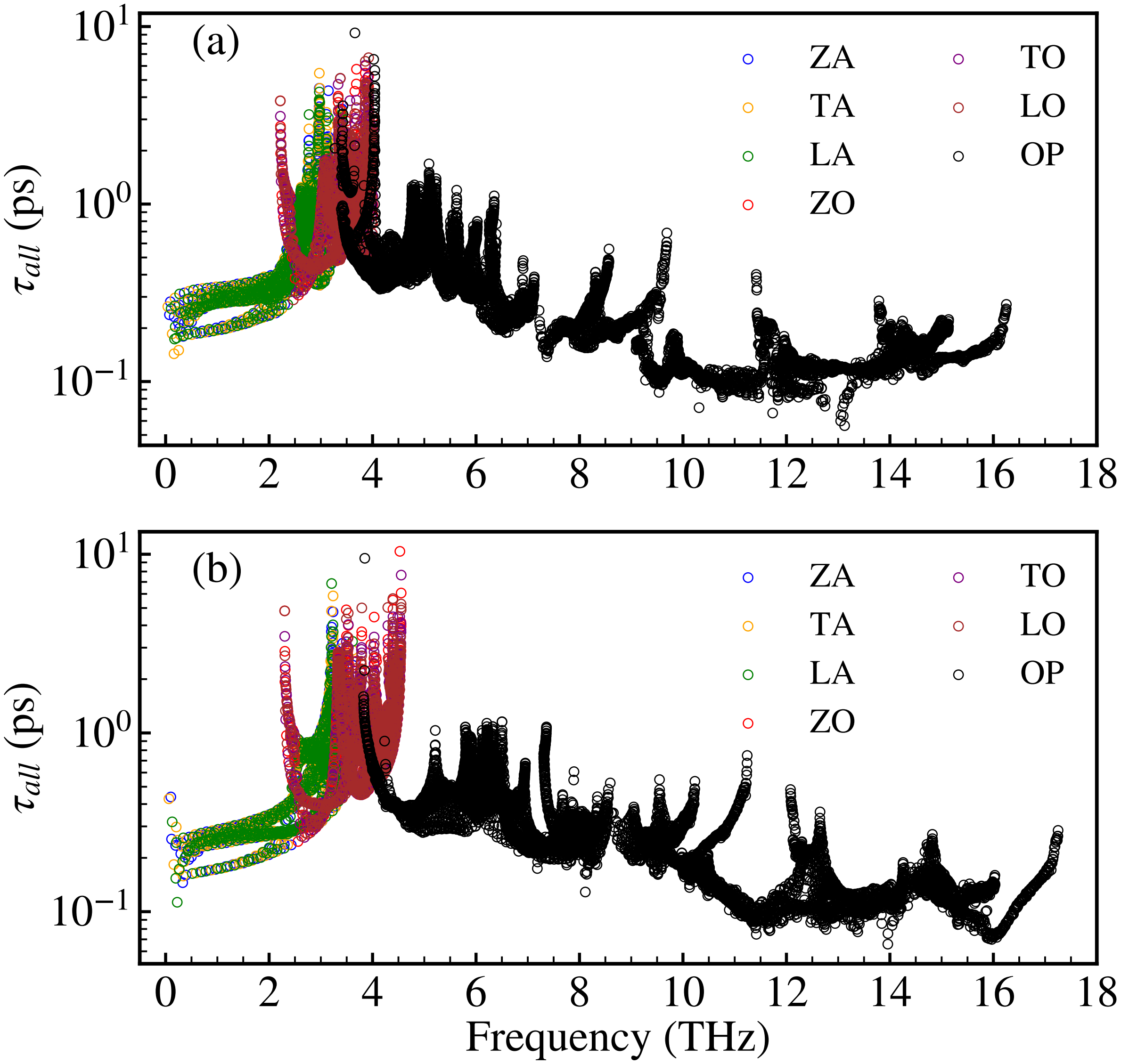

Figure S9 Relaxaton time including $\tau_\lambda^{\text{anhar}}+\tau_\lambda^{\text{iso}}+\tau_\lambda^{\text{4ph}}$ as function of frequency for (a) $ScYCBr_2$ and (b) Sc_2CBr_2. The plot also shows the contribution of each phonon mode.

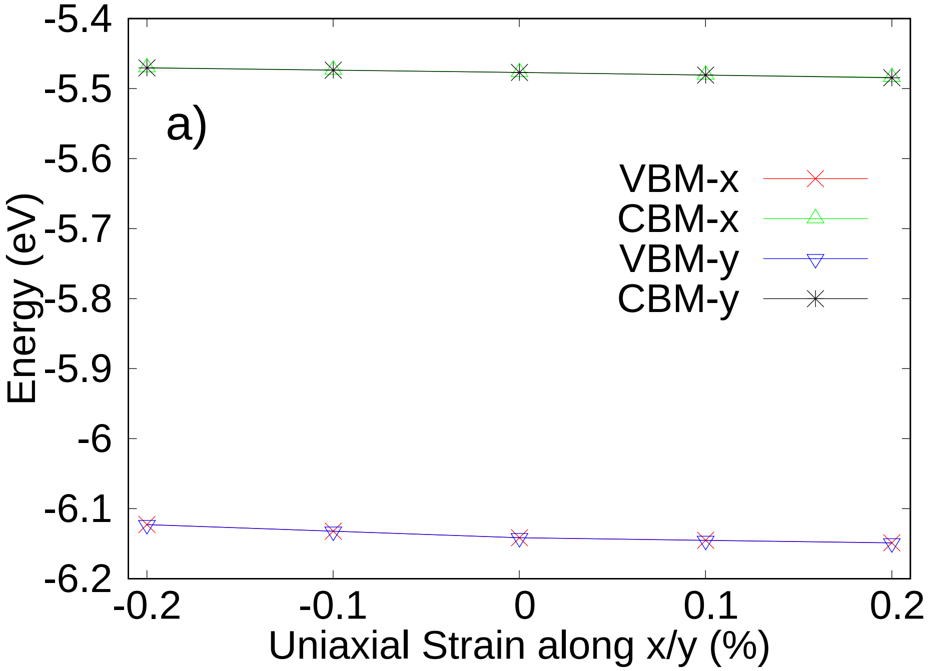

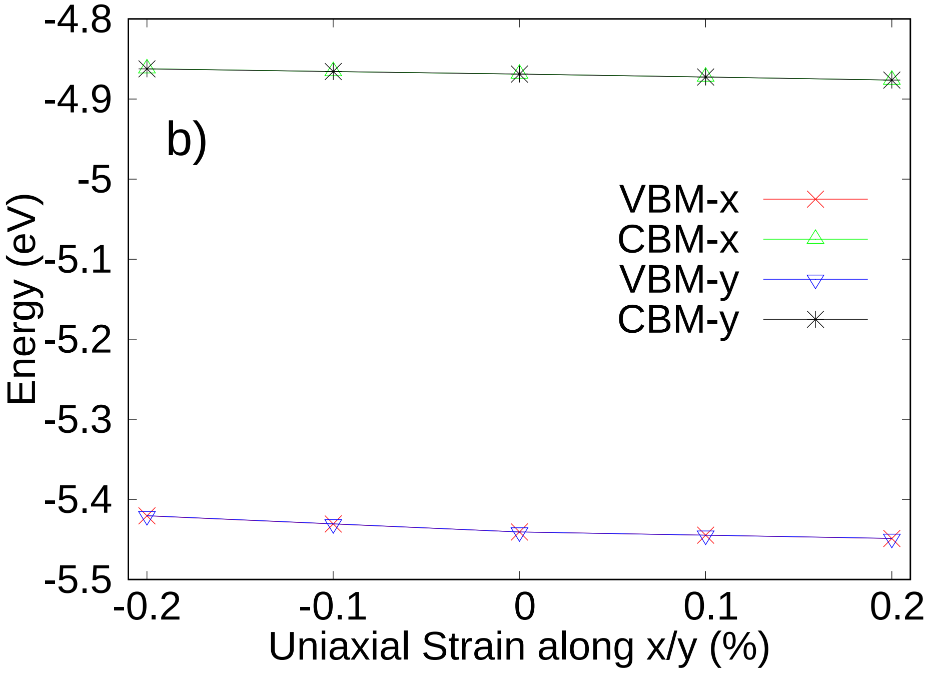

Figure S10 Variation of band-edge energies versus uni-axial strain of (a) $ScYCBr_2$ and (b) Sc_2CBr_2. The fits are represented by solid lines.

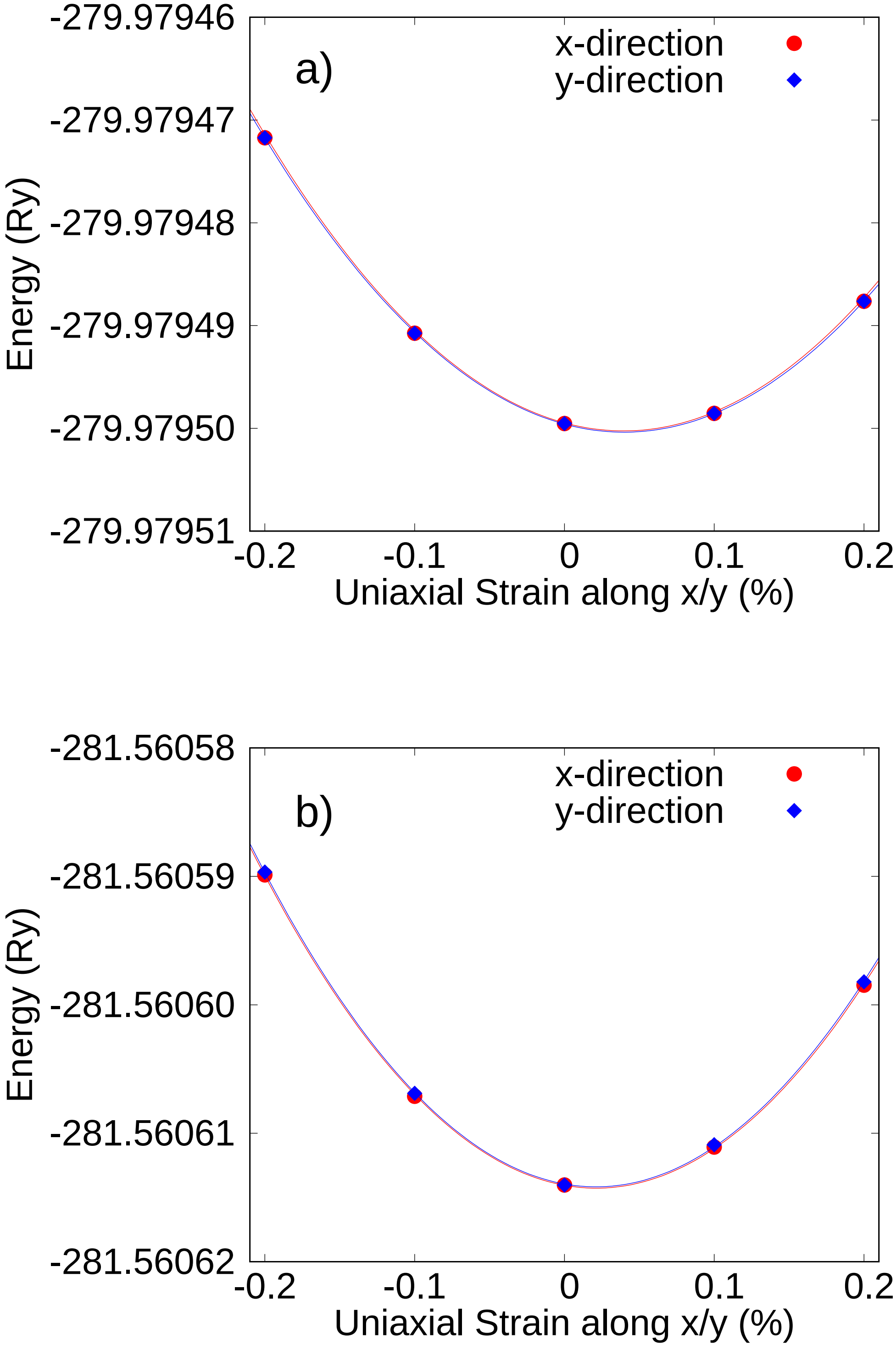

Figure S11 Variation of strain energy versus uni-axial strain for (a) $ScYCBr_2$ and (b) Sc_2CBr_2. The fits are represented by solid lines.

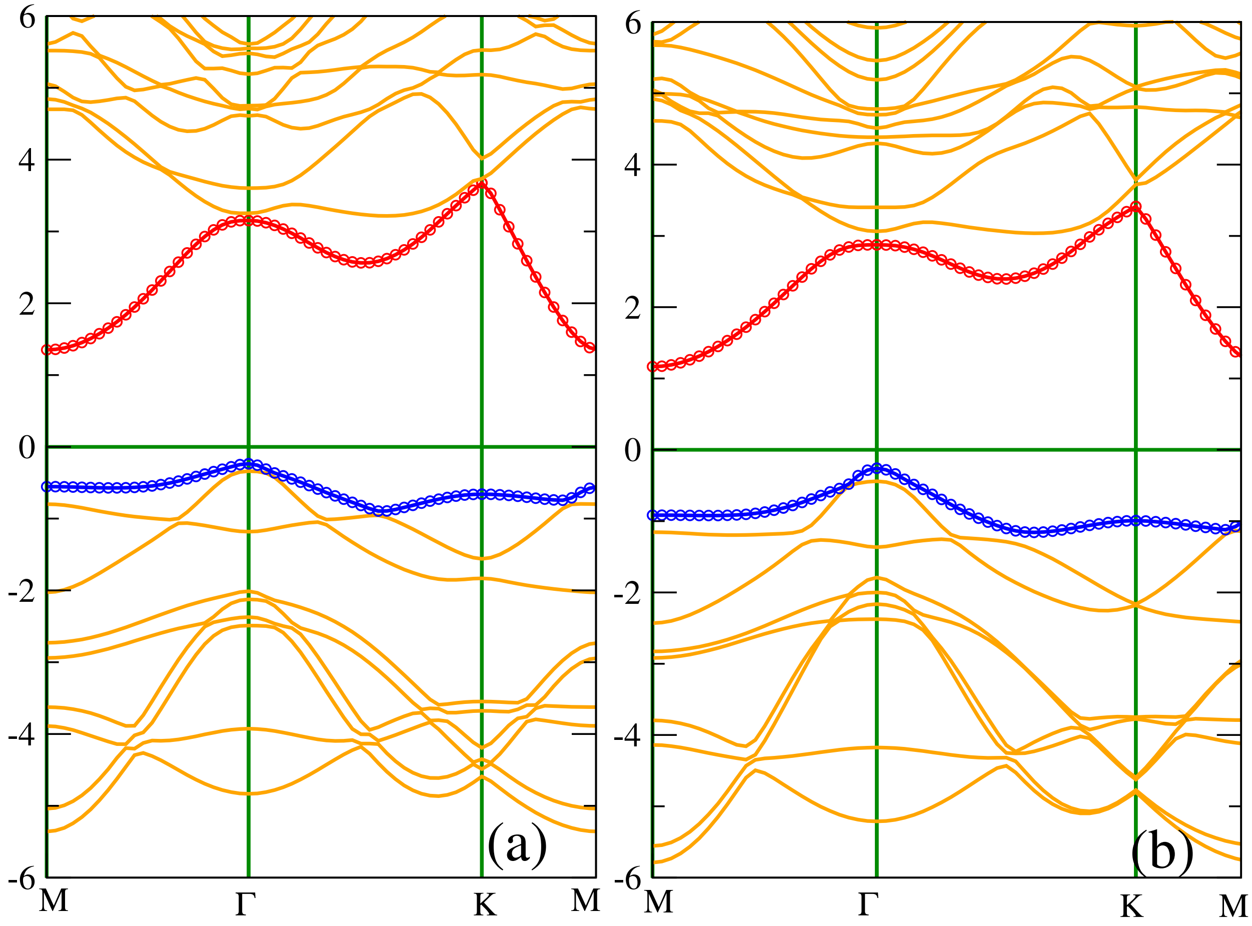

Figure S12 Band structure of (a) $ScYCBr_2$ and (b) Sc_2CBr_2 calculated using the HSE06 functional. The LUMO and HOMO levels are highlighted with blue and red color, respectively. The calculated band-gap values for $ScYCBr_2$ and Sc_2CBr_2 are 1.56 and 1.42 eV, respectively.

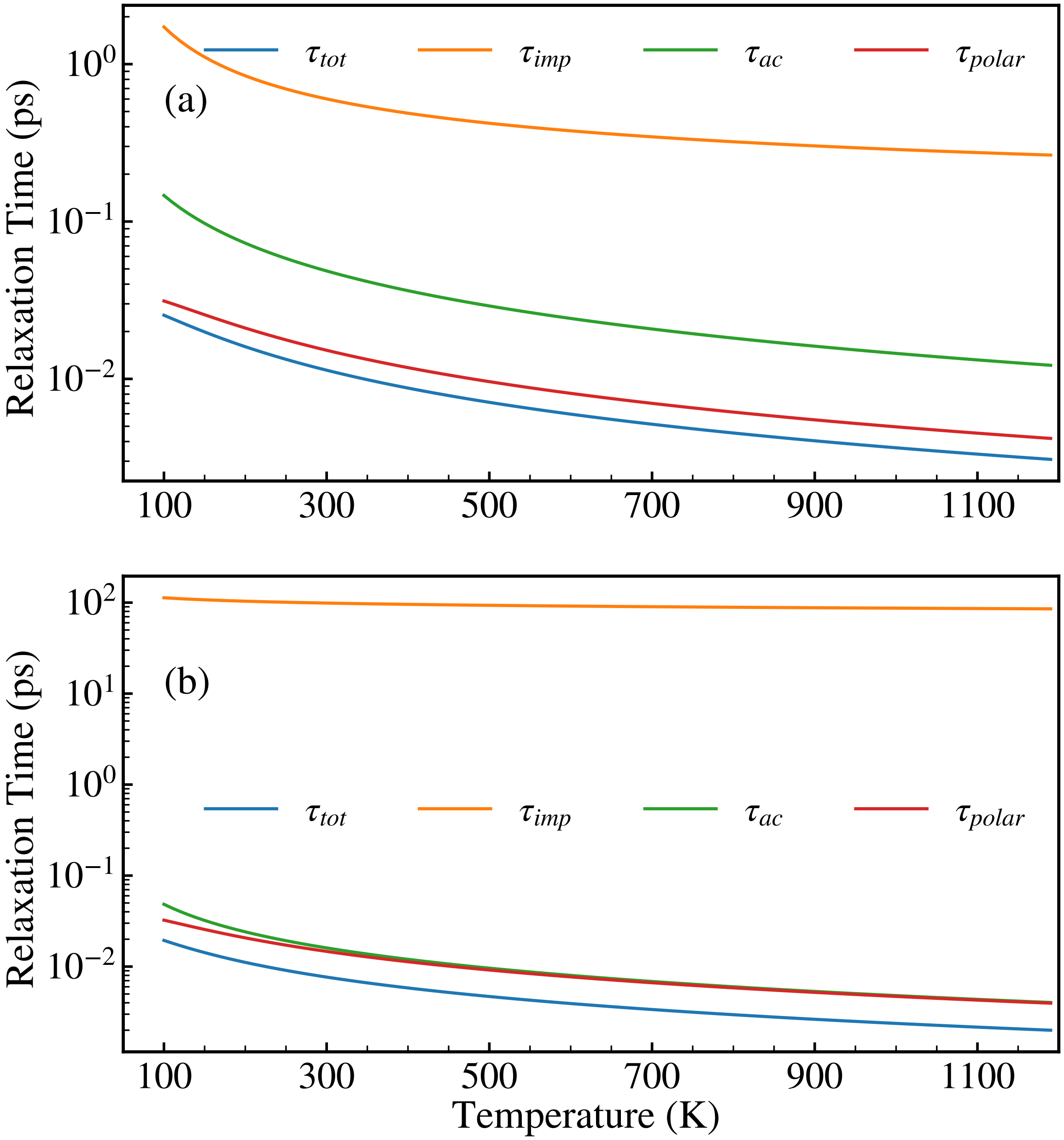

Figure S13 Relaxation time τ of (a) $ScYCBr_2$ and (b) Sc_2CBr_2 vs temperature. To improve the constant-time approximation, we adopted a full-relaxation time model that depends on the temperature T. Here (*tot*), (*pol*), (*ac*), and (*imp*) stands respectively for total, polar-optical phonon scattering rate, acoustic phonon scattering rate, and impurity scattering rate. For more information see Ref.[15]

References

(1) S. Meziane, H. Feraoun, T. Ouahrani, C. Esling Effects of Li and Na Intercalation on Electronic, Bonding, and Thermoelectric Transport Properties of MX_2 (M= Ta; X= S or Se) Dichalcogenides–Ab initio Investigation *J. Alloys Compd.*, **2013** *581*, 731-740

(2) M. Born and K. Huang, Dynamical Theory of Crystal Lattices, Clarendon, Oxford, 1954

(3) Ouahrani, T.; Boufatah, R. M. Understanding the Semiconducting-To-Metallic Transition in the CF_2Si Monolayer under Shear Tensile Strain. *Crystals* **2022**, *12*, 1476.

(4) Luo, Y.; Cheng, C.; Chen, H.-J.; Liu, K.; Zhou, X.-L. Systematic Investigations of the Electron, Phonon and Elastic Properties of Monolayer M2C (M = v, Nb, Ta) by First-Principles Calculations. *J. Phys.: Condens. Matter* **2019**, *31*, 405703.

(5) Cooper, R. C.; Lee, C.; Marianetti, C. A.; Wei, X.; Hone, J.; Kysar, J. W. Nonlinear Elastic Behavior of Two-Dimensional Molybdenum Disulfide. *Phys. Rev. B* **2013**, *87*, 035423.

(6) Vu, T. V.; Kartamyshev, A. I.; Hieu, N. V.; Dang, T. D. H.; Nguyen, S.-N.; Poklonski, N. A.; Nguyen, C. V.; Phuc, H. V.; Hieu, N. N. Structural, Elastic, and Electronic Properties of Chemically Functionalized Boron Phosphide Monolayer. *RSC Adv.* **2021**, *11*, 8552–8558.

(7) M. R. Hermes and S. Hirata, First-order Dyson Coordinates and Geometry *J. Phys. Chem. A* **2013**, *117*, 7179-7189.

(8) Masuki, R.; Nomoto, v; Arita, R.; Tadano, T.; Full Optimization of Quasiharmonic Free Energy with an Anharmonic Lattice Model: Application to Thermal Expansion and Pyroelectricity of Wurtzite GaN and ZnO, *Phys. Rev. B* **2023**, *107*, 134119.

(9) Tran, T.-A.; Hai, L. S.; Vi; Nguyen, C. Q.; Nghiem, N. T.; Le; Hieu, N. N. Janus Struc-

tures of the C_2h Polymorph of Gallium Monochalcogenides: First-Principles Examination of Ga_2XY (X/Y = S, Se, Te) Monolayers. *RSC Adv.* **2023**, *13*, 12153–12160.

(10) Jacoboni, C. Theory of Electron Transport in Semiconductors: A Pathway from Elementary Physics to Nonequilibrium Green Functions, Vol. 165 (Springer Science Business Media, 2010).

(11) Madsen, G. K. Singh, D. J. BoltzTraP. A code for Calculating Band-structure Dependent Quantities. *Comp. Phys. Commun.* **2006** *175*, 67–71.

(12) Farris, R.; Maccioni, M. B.; Filippetti, A. Fiorentini, V.; Theory of Thermoelectricity in Mg_3Sb_2 with an Energy-and Temperature- Dependent Relaxation Time. *J. Phys. Condens. Matter* **2018**, *31*, 065702.

(13) Ridley, B. Polar-optical-phonon and electron-electron Scattering in Large-bandgap Semiconductors. *J. Phys. Condens. Matter* **1998**, *10*, 6717-6726.

(14) Jayaraj, A.; Siloi, I.; Fornari, M.; Nardelli, M. B. Relaxation Time Approximations in PAOFLOW 2.0 *Sci. Rep.* **2022**, *12*, 4993.

(15) Casu, G.; Bosin, A.; Fiorentini, V.; Efficient Thermoelectricity in $Sr_2Nb_2O_7$ With Energy-dependent Relaxation Times, *Phys. Rev. Mat.* **2020**, *4*, 075404.
